# NELF‐A controls *Drosophila* healthspan by regulating heat‐shock protein‐mediated cellular protection and heterochromatin maintenance

**DOI:** 10.1111/acel.13348

**Published:** 2021-03-31

**Authors:** Zhen‐Kai Ngian, Wei‐Qi Lin, Chin‐Tong Ong

**Affiliations:** ^1^ Temasek Life Sciences Laboratory National University of Singapore Singapore Singapore; ^2^ Department of Biological Sciences National University of Singapore Singapore Singapore

**Keywords:** *Drosophila*, healthspan, heat‐shock proteins, heterochromatin, *Nelf‐A*, stress responses

## Abstract

NELF‐mediated pausing of RNA polymerase II (RNAPII) constitutes a crucial step in transcription regulation. However, it remains unclear how control release of RNAPII pausing can affect the epigenome and regulate important aspects of animal physiology like aging. We found that NELF‐A dosage regulates *Drosophila* healthspan: Halving NELF‐A level in the heterozygous mutants or via neuronal‐specific RNAi depletion improves their locomotor activity, stress resistance, and lifespan significantly. Conversely, *NELF‐A* overexpression shortens fly lifespan drastically. Mechanistically, lowering NELF‐A level facilitates the release of paused RNAPII for productive transcription of the heat‐shock protein (*Hsp*) genes. The elevated HSPs expression in turn attenuates the accumulation of insoluble protein aggregates, reactive oxidative species, DNA damage and systemic inflammation in the brains of aging *NELF‐A* depleted flies as compared to their control siblings. This pro‐longevity effect is unique to NELF‐A due to its higher expression level and more efficient pausing of RNAPII than other NELF subunits. Importantly, enhanced resistance to oxidative stress in *NELF‐A* heterozygous mutants is highly conserved such that knocking down its level in human SH‐SY5Y cells attenuates hydrogen peroxide‐induced DNA damage and apoptosis. Depleting NELF‐A reconfigures the epigenome through the maintenance of H3K9me2‐enriched heterochromatin during aging, leading to the repression of specific retrotransposons like *Gypsy*‐*1* in the brains of *NELF*‐*A* mutants. Taken together, we showed that the dosage of neuronal *NELF*‐*A* affects multiple aspects of aging in *Drosophila* by regulating transcription of *Hsp* genes in the brains, suggesting that targeting transcription elongation might be a viable therapeutic strategy against age‐onset diseases like neurodegeneration.

## Graphical abstract

NELF‐A mediates RNA polymerase II pausing at many stimuli‐responsive genes in metazoans. Halving NELF‐A dosage in *Drosophila* facilitates productive transcription of heat‐shock protein (Hsp) genes. The elevated level of HSPs attenuates protein aggregation and reduces ROS‐induced oxidative DNA damage. This in turn suppresses systemic inflammation and promotes heterochromatin‐mediated maintenance of genome integrity, leading to extended animal healthspan.

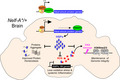

**Abbreviations**
HSP: heat‐shock protein
NELF: negative elongation factor
RNAPII: RNA polymerase II
ROS: reactive oxygen species

## 1 INTRODUCTION

Controlled release of paused RNA polymerase II (RNAPII) at the promoter‐proximal region is one of the most critical regulatory steps in transcription (Chen et al., 2018; Core & Adelman, 2019). Yet how it may influence important aspects of animal physiology like aging remains unclear. RNAPII pausing occurs at a huge fraction of eukaryotic genes (Core et al., 2008; Min et al., 2011) and is mediated by the functionally conserved negative elongation factor (NELF) complex comprised of four subunits (A, B, C/D, and E; Narita et al., 2003; Wu et al., 2005). NELF complex interacts with 5,6‐dichloro‐1‐β‐d‐ribobenzimidazole sensitivity‐inducing factor (DSIF) and the hypophosphorylated form of RNAPII to induce its pausing at ~30–60 nucleotides downstream of the transcriptional start site (TSS; Wada et al., 1998; Wu et al., 2003; Yamaguchi et al., 1999). Phosphorylation of NELF complex and RNAPII by the positive transcription elongation factor‐b (P‐TEFb) evicts NELF complex, thereby releasing RNAPII for productive elongation (Lis et al., 2000).

Interestingly, perturbations of different NELF subunits impact transcriptional output and expression patterns in cell‐types specific manner (Gilchrist et al., 2008; Wang et al., 2010). In vivo studies showed that NELF‐mediated promoter‐proximal stalling of RNAPII ensures robust and coordinated gene activation in response to different types of signals and external stimuli (Gilchrist et al., 2010, 2012; Saunders et al., 2013; Williams et al., 2015). In *Drosophila* S2 cells and larvae, genes affected by *NELF* depletion are enriched for heat‐shock and immune responses (Gilchrist et al., 2008, 2012). As many of these genes are linked to the process of aging, it is plausible that NELF may play a role in regulating animal lifespan.

We found that the level of NELF‐A, but not other subunits, regulates adult fitness and lifespan in fly. Both heterozygous *NELF*‐*A* mutants and neuronal‐depleted *NELF*‐*A* flies exhibited increased locomotor activity and lifespan as compared to their control siblings. Halving the level of NELF‐A reduces RNAPII pausing at heat‐shock protein (*Hsp*) genes, leading to significant upregulation of their expression. Elevated level of HSPs in the brain and head tissues suppresses the accumulation of protein aggregates and reactive oxygen species (ROS), attenuates systemic inflammation, and enhances animal resistance against external oxidative stress during aging. Similarly, we observed enhanced stress resistance in human cells depleted of *NELF*‐*A*, whose N‐ and C‐terminal region is highly conserved to *Drosophila* NELF‐A protein (Wu et al., 2005). In heterozygous *NELF*‐*A* mutant flies, reduced DNA damage and increased S‐adenosyl methionine (SAM) concentration coincide with the maintenance of the repressive H3K9me2‐marked heterochromatin and the repression of specific classes of TEs during aging. Taken together, NELF‐A‐mediated RNAPII pausing influences animal healthspan by regulating HSP‐mediated cellular protection and H3K9me2‐dependent maintenance of genome integrity.

## 2 RESULTS

### 2.1 Neuronal expression of *NELF*‐*A* regulates lifespan and locomotor activity

**FIGURE 1:**
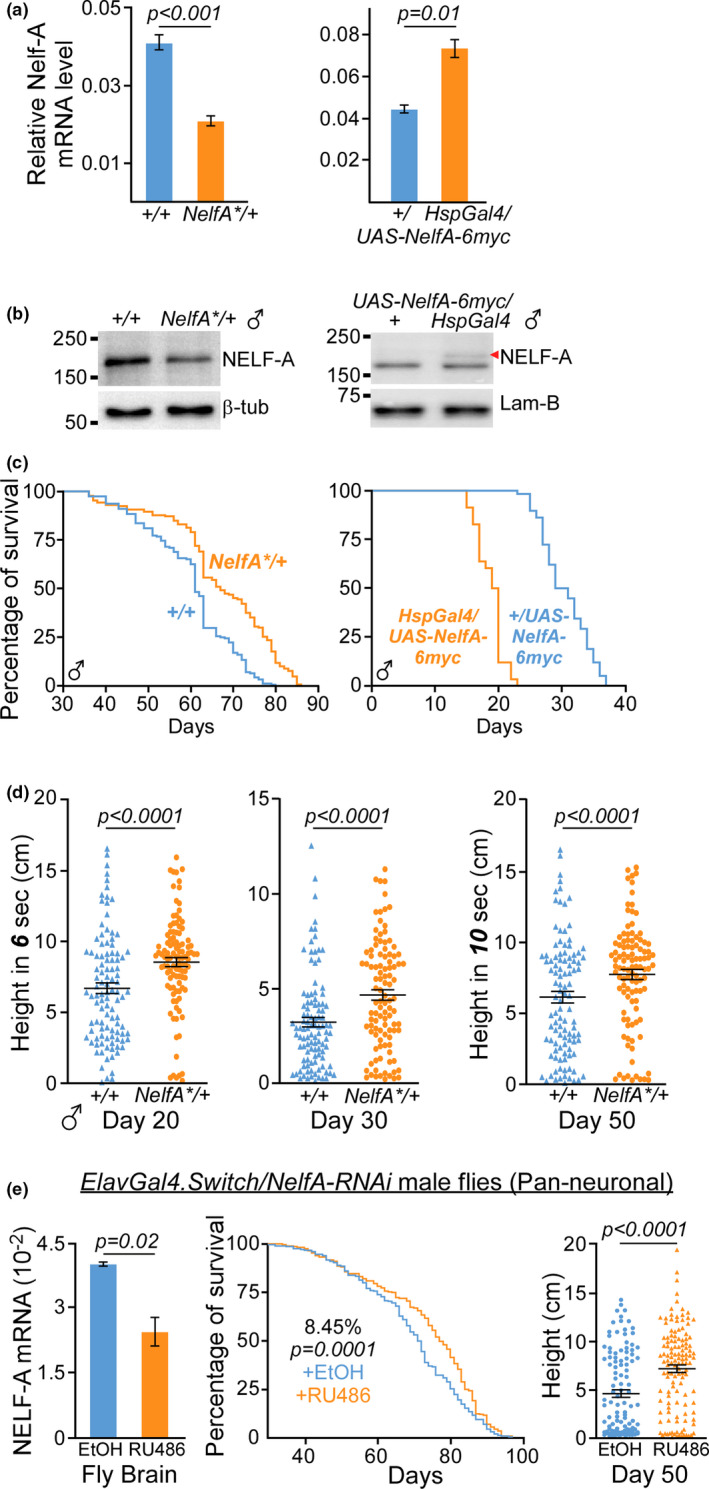
Lower neuronal level of *NELF*‐*A* improves animal lifespan and locomotor activity. (a) Quantification of *NELF*‐*A* mRNA in NelfA*/+ (left) and *HspGal4*/*UAS*‐*NelfA*‐*6myc* (right) fly heads as compared to their respective control siblings. Data presented as mean ± SEM, *n* = 3, unpaired two‐tailed *t* test. (b) Immunoblot of endogenous and myc‐tagged (red arrowhead) NELF‐A protein in the heads harvested from male NelfA*/+ flies (left) and *Hsgal4*/*UAS*‐*NelfA*‐*6myc* flies (right), respectively. β‐tub: β‐tubulin, Lam‐B: Lamin‐B1. (c) Lifespan assay of male NelfA*/+ flies (9.8%, *n* = 180, *p* < 0.0001; left) and *HspGal4*/*UAS*‐*NelfA*‐*6myc* flies (−44.1%, *n* = 160, *p* < 0.0001; right) as compared to their control siblings. Log‐rank test. (d) Climbing assay of male NelfA*/+ flies and their control siblings at different ages (D20: 38.8%; D30: 52.9%; D50: 34.5%, *n* = 105 per group). Percent calculated based on median height, Mann–Whitney test. (e) Pan‐neuronal *NELF*‐*A* depletion in RU486‐treated *ElavGal4*. *Switch*/*NelfA*‐*RNAi* male flies improved lifespan and locomotor activity. Left: Quantification of *NELF*‐*A* mRNA in the brains harvested from EtOH or RU486 treated flies. Data presented as mean ± SD, *n* = 2, unpaired two‐tailed *t* test. Middle: Lifespan assay (8.45%, *n* = 280 flies after combining two independent cohorts, *p* = 0.0001, Log‐rank test). Right: Climbing assay (*n* = 100–120 flies, Mann–Whitney test)

To examine the regulatory roles of *NELF* genes in *Drosophila* lifespan, we first examined the highly expressed *NELF*‐*A* gene and backcrossed heterozygous mutant *Nelf*‐*A^KG09483^*/*Tm3* line (Chopra et al., 2009; Tsai al., 2016; Wang et al., 2010) to *w^1118^* male for at least 10 consecutive generations. Compared to their control siblings, backcrossed heterozygous male *Nelf*‐*A^KG09483^*/+ (herein NelfA*/+) flies expressed less than half the level of *NELF*‐*A* mRNA (Figure 1a) and protein (Figures 1b and S1A,B). Both the male and female NelfA*/+ flies showed approximately 10% and 20% increase in their lifespan, respectively (Figures 1c and S1C), suggesting that animal longevity may be affected by *NELF*‐*A* expression level. To test this, we ectopically expressed low level of NELF‐A protein by crossing *UAS*‐*NelfA*‐*6myc* line to *Hsp70*‐*Gal4* driver. When cultured at 30°C, their F1 offspring expressed higher level of *NELF*‐*A* mRNA (Figure 1a), produced exogenous myc‐tagged NELF‐A protein (Figures 1b and S1A), and had significantly shorter lifespan than their control siblings (Figures 1c and S1C). This indicates that the level of *NELF*‐*A* expression is inversely correlated to animal lifespan.

*Drosophila* undergoes age‐dependent decline in their locomotor activity (Jones & Grotewiel, 2011). Negative geotaxis assays showed that heterozygous NelfA*/+ male flies consistently exhibited faster locomotor activity than their control siblings at different ages in adulthood (Figure 1d), suggesting that lowering the level of *NELF*‐*A* expression may improve overall fitness or healthspan of the animals.

We next used *GeneSwitch* (*GS*) system to determine the organs by which *NELF* genes may regulate animal healthspan. As depletion of *NELF* genes affects the expression of immune‐response genes in *Drosophila* S2 cells (Gilchrist et al., 2012), we examined the effect of RNAi‐mediated depletion of *NELF* gene in the fat bodies from the brain (*Bun*‐*GS*) and abdomen (*106*‐*GS*; Hwangbo et al., 2004). In addition, *NELF* genes were also knockdown (KD) in the neurons using *Elav*‐*GS* line given that NF‐κB immune signaling in the brain regulates *Drosophila* lifespan (Kounatidis et al., 2017). To mimic the heterozygous NelfA*/+ flies, the level of RU486 administrated in fly food was optimized to induce ~50% KD of *NELF*‐*A* expression in the brain (Figure 1e). Pan‐neuronal RNAi KD of *NELF*‐*A* gene led to modest yet statistically significant increase in the lifespan of male flies (~8%–10%; Figures 1e and S1D) and their locomotor activity (Figure 1e). On the other hand, knocking down of *NELF*‐*A* expression in the fat bodies in the brain or abdomen has no statistically significant effect on adult lifespan (Figure S1E,F). This suggests that the improved longevity and locomotor activity observed in heterozygous NelfA*/+ flies may be largely attributed to the regulatory roles of NELF‐A in the neuronal populations. To ascertain if other *NELF* subunits may exert similar effect as *NELF*‐*A*, *Elav*‐*GS* line was used to KD *NELF*‐*B* and *NELF*‐*E* genes. Surprisingly, despite substantial reduction in the level of *NELF*‐*B* and *NELF*‐*E* expression by tissue‐specific RNAi KD, the male flies did not show significant change in their adult lifespan as compared to the control siblings (Figure S1G). Similarly, overexpression of *UAS*‐*Nelf*‐*E*‐*6myc* gene by *Hsp70*‐*Gal4* driver also did not reduce their lifespan as seen with the *NELF*‐A overexpressing flies (Figure S1H). Taken together, these results suggest that the healthspan of *Drosophila* is highly dependent on the expression level of *NELF*‐*A*, rather than other *NELF* subunits, within the neuronal populations.

### 2.2 *NELF‐A* reduction leads to pro‐longevity transcriptional signatures

**FIGURE 2:**
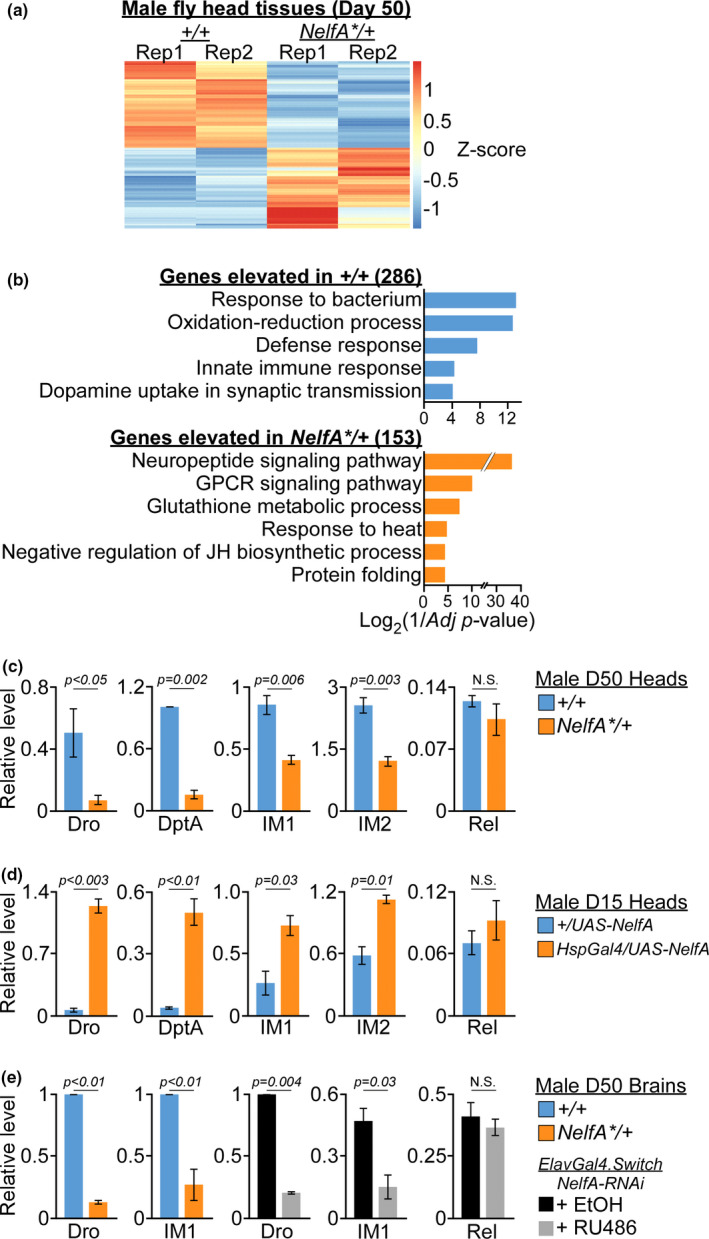
Pro‐longevity transcriptome in *NELF*‐*A* depleted fly heads. (a) Heatmap of differentially expressed genes (DEGs) in the heads of D50 male NelfA*/+ flies and their +/+ control siblings. Rep: Biological replicate. (b) GO analysis of DEGs identified in NelfA*/+ and their control siblings fly heads (adjusted *p* < 0.05, with Benjamini‐Hochberg correction). (c) RT‐PCR validation in the heads of D50 NelfA*/+ flies and their control siblings. Data presented as mean ± SEM, *n* = 3 biological replicates, unpaired two‐tailed *t* test. (d) Quantification of *AMP* genes expression in the heads of D15 *HspGal4*/*UAS*‐*NelfA*:*6myc* flies and their control siblings. Data presented as mean ± SD, *n* = 2 biological replicates, unpaired two‐tailed *t* test. (e) Quantification of *AMP* genes expression in the brains isolated from either male NelfA*/+ flies or pan‐neuronal *NELF*‐*A* KD flies (RU486‐treated *ElavGal4*. *Switch*/*NelfA*‐*RNAi*) and their respective control siblings. Data presented as mean ± SD, *n* = 2 biological replicates, unpaired two‐tailed *t* test

To identify downstream molecular pathways that may be responsible for the improved healthspan observed in NelfA*/+ flies, RNA‐sequencing was performed on the mRNA isolated from the heads of Day 50 (herein D50) male heterozygous mutants and their control siblings. Differential gene expression analysis using DESeq2 package revealed two cluster of genes that were highly elevated in either control siblings (+/+) or heterozygous mutants (NelfA*/+; Figure 2a, Table S1). Gene ontology (GO) analysis of the differentially expressed genes in the *Drosophila* heads revealed transcriptional changes in several stimulus‐responsive genes that resembled *NELF*‐*B*/*E* depleted S2 cells (Gilchrist et al., 2008, 2012; Table S2). Like *NELF*‐*B*/*E* depleted S2 cells, NelfA*/+ flies had reduced expression in genes that are enriched for defense response and response to bacterium (Figure 2b). In addition, the NelfA*/+ mutants also showed upregulation of genes that are involved in different signaling pathways, heat‐shock response, and protein folding (Figures 2b and S2).

During aging, elevated expression of immune‐response pathway genes such as the induced immune molecules (IM) and antimicrobial peptides (AMPs) may trigger excessive inflammation that leads to morbidity in older flies (Badinloo et al., 2018; Cao et al., 2013; Kounatidis et al., 2017). In accordance, overexpression of Rel (NF‐κB) transcription factor can induce *AMP* genes in the absence of upstream signals; whereas reducing Rel level may attenuate inflammation and promote longevity (Kounatidis et al., 2017). Consistent with these findings, there was a significant increase in the expression of *Rel* and *AMP* (*AttC*, *DptA*) genes in *w^1118^* male flies during aging (Figure S2A). In contrast, elevated expression of genes involved in heat‐shock responses and protein refolding has been well‐documented to promote animal health and longevity (Biteau et al., 2010; Morrow et al., 2004; Wang et al., 2004).

The dichotomy in the expression of immune‐response and heat‐shock response genes led us to investigate if their transcription is directly regulated by NELF‐A in the *Drosophila* heads. The lower expression of *AMP* (*Dro*, *DptA*), *IM1*, and *IM2* genes in the heads of NelfA*/+ flies as compared to their control siblings was first validated by RT‐PCR (Figures 2c and S2B). Conversely, ectopic expression of *NELF*‐*A* caused the upregulation of *AMP* and *IM* genes in heads of *HspGal4*/*UAS*‐*NelfA*‐*6myc* flies as compared to their control siblings (Figures 2d and S2C). These results suggest that the level of *NELF*‐*A* is positively correlated to the expression of *AMP* and *IM* genes in the head of the flies. To determine the tissue‐specific role played by NELF‐A, D50 brains from both NelfA*/+ and *ElavGal4*. Switch/*NelfA*‐RNAi flies were dissected and analyzed by RT‐PCR. Like the NelfA*/+ flies, neuronal *NELF*‐*A* KD by RU486 treatment led to reduced *Dro* and *IM1* expression in the brains as compared to ethanol (EtOH) treated control siblings (Figures 2e and S2D). This indicates that neuronal NELF‐A can regulate the expression of *AMP* and *IM* genes in the brains. In S2 cells, *NELF*‐*B*/*E* depletion reduces the transcription of *Rel* and concomitant downregulation of *AMP* genes (Gilchrist et al., 2012). As *Rel* expression is relatively similar between NelfA*/+ flies and their control siblings (Figure 2c,e), NELF‐A is likely to affect AMPs level indirectly or via distinct mechanism in the fly heads.

Elevated AMPs level can cause neurodegeneration in old adult fly brains, marked by increased number of vacuoles in the brain sections (Cao et al., 2013; Kounatidis et al., 2017). In accordance, vacuoles (red arrow) were observed in D50 but not D20 *w^1118^* fly brains (Figure S2E). Since climbing ability of older flies is impaired by age‐dependent neurodegeneration (Kounatidis et al., 2017), we asked if the improved locomotor activity in NelfA*/+ flies was due to reduced AMPs level, and hence lower neurodegeneration as compared to their control siblings. Histological examination of D50 brains harvested from *NELF*‐A*/+, RU486‐treated *ElavGal4*. *Switch*/*NelfA*‐*RNAi* and their respective control sibling flies did not show any morphological difference (data not shown). This unexpected observation might be explained by the significantly lower level of *Rel* and *AMPs* expression in NelfA*/+ and their control siblings as compared to age‐matched *w^1118^* flies (Figure S2A). Nevertheless, the lower *AMPs* expression in NelfA*/+ flies is indicative of less systemic inflammation (Figure 2b,c), presumably attributed to other pro‐longevity molecular changes.

### 2.3 *NELF* depletion induces HSPs to protect against oxidative stress

**FIGURE 3:**
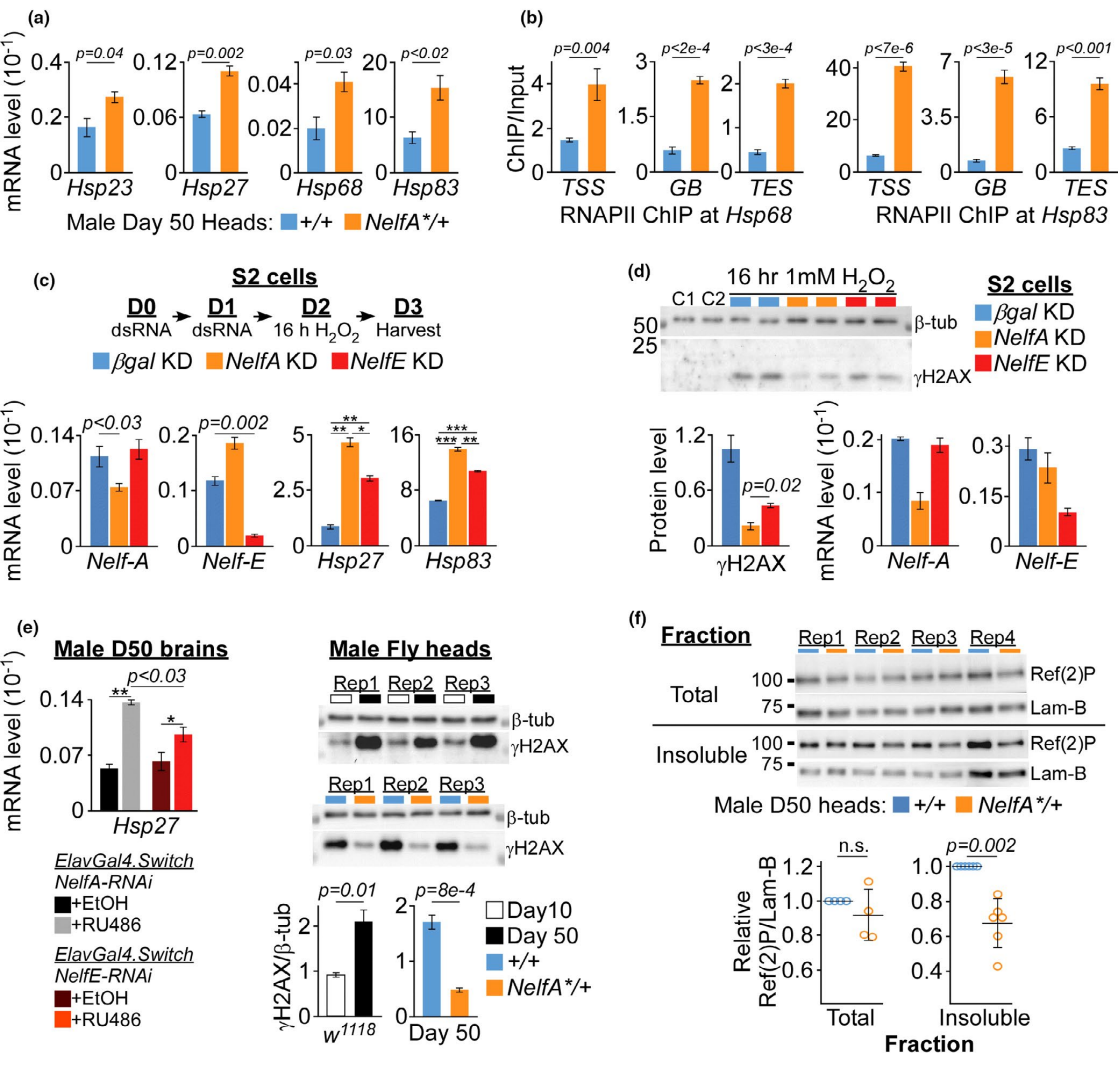
Elevated HSPs enhances stress resistance and protein homeostasis. (a) Quantification of *Hsp* genes expression in the heads of D50 male NelfA*/+ flies and their control siblings. Data presented as mean ± SEM, *n* = 3, unpaired two‐tailed *t* test. (b) RNAPII occupancy at the TSS, GB, and TES of *Hsp68* and *Hsp83* in the heads of D50 NelfA*/+ flies and their control siblings. Representative ChIP‐data presented as mean ± SD from triplicate qPCR reactions. Unpaired two‐tailed *t* test. Data of another biological replicate are presented in Figure S3. (c) Top: *NELF* genes were KD by dsRNA on Day 0 and 1 (D0, D1) followed by 16 h of 5 mM H_2_O_2_ treatment. Bottom: Quantification of mRNA expression on D3 with data presented as mean ± SD, *n* = 2. *p = 0.005; **p < 0.001 and ***p < 0.0003, unpaired one‐tailed *t* test. (d) *βgal*, *NELF*‐*A* or *NELF*‐*E* KD S2 cells treated with 1 mM H_2_O_2_ were harvested for γ‐H2AV immunoblot (Top) and RT‐PCR of *NELF* genes (Bottom). The level of γ‐H2AV was normalized to β‐tubulin and presented as mean ± SD, *n* = 2, unpaired two‐tailed *t* test. (e) Quantification of *Hsp27* expression in brains isolated from pan‐neuronal *NELF*‐*A* and *NELF*‐*E* KD flies as compared to their respective control siblings. Data presented as mean ± SD, *n* = 2. *p < 0.03; **p < 0.003, two‐tailed *t* test. (f) Top: Immunoblot of γ‐H2AV in heads of aging *w^1118^*, NelfA*/+ flies and their control siblings. Rep: biological replicate. Bottom: Data presented as mean ± SD, *n* = 3, unpaired two‐tailed *t* test. (g) Top: Immunoblot of Ref(2)P in total lysates and insoluble fraction of the heads harvested from D50 NelfA*/+ flies and their control siblings. Bottom: Graphs of individual and mean (±SD) level of total and insoluble Ref(2)P fractions. Paired two‐tailed *t* test

HSPs are molecular chaperones that regulate protein homeostasis in response to environmental stress (Richter et al., 2010). In accordance, ectopic expression of specific *Hsp* genes could lead to extension in *Drosophila* lifespan (Morrow et al., 2004; Wang et al., 2004). This suggests that the elevated expression of *Hsp* genes may promote the healthspan of NelfA*/+ flies. RT‐PCR was first used to validate their expression genes in male fly heads (Figure 3a) and brains (Figure S3A). The presence of specific DNA elements, including GAGA factor binding site, initiates the recruitment and pausing of RNAPII at the promoters of *Hsp* genes (Core & Adelman, 2019). It is plausible that reduced NELF‐A level in NelfA*/+ flies (Figures 1b and S1A) may facilitate the release of paused RNAPII at *Hsp* promoters, leading to higher transcriptional output that is similar to *NELF*‐depleted S2 cells (Gilchrist et al., 2008). To test this, RNAPII chromatin immunoprecipitation (ChIP) was performed on D50 heads harvested from NelfA*/+ flies and their control siblings. Site‐specific primers on *Hsp68* and *Hsp83* genes were designed based on RNAPII‐ChIP‐seq data from KC167 cells (Figure S3B,C). IgG‐ChIP yielded undetectable signal whereas opposite patterns of RNAPII‐ChIP signals were observed at the highly transcribed *Rpl32* gene and silenced *Het3R2* region (Figure S3D). Compared to control siblings, there was significantly higher level of RNAPII enrichment at >100 nucleotides downstream of TSS, in the gene body (GB) and at the transcriptional end site (TES) of *Hsp68* and *Hsp83* genes in NelfA*/+ fly heads (Figures 3b, S3E,F). This result is consistent with the notion that lower NELF‐A protein level releases paused RNAPII for more productive transcription of *Hsp68* and *Hsp83* genes.

In S2 cells, RNAi KD of different *NELF* subunits led to increased expression of many *Hsp* genes (Gilchrist et al., 2008). The possibility of HSPs acting downstream of NELF‐A led us to question why genetic manipulation of only *NELF*‐*A*, but not other *NELF* subunits, affects animal lifespan (Figures S1G & S1H). To this end, we examined the effect of depleting either *NELF*‐*A* or *NELF*‐*E* gene in S2 cells which were then exposed to hydrogen peroxide (H_2_O_2_) treatment (Figure 3c). Double‐stranded (ds) RNA‐mediated KD of *NELF*‐*A* or *NELF*‐*E* gene led to significant reduction in their mRNA expression as compared to *βgal* KD cells (Figure 3c). Interestingly, depletion of both *NELF* genes led to elevated expression of *Hsp27* and *Hsp83* genes upon H_2_O_2_ treatment, although the level of *Hsp* genes was significantly higher in *NELF*‐*A* KD cells (Figure 3c). This suggests that *NELF*‐*A* might play a more dominant role in RNAPII pausing as compared to *NELF*‐*E*, such that its depletion can cause higher *Hsp* expression and thus better responses against oxidative stress. To test this, the level of DNA double‐stranded breaks caused by H_2_O_2_ treatment was measured by γ‐H2AV antibody. In contrast to untreated control S2 cells (C1 and C2), 16 hours of incubation with 1 mM H_2_O_2_ triggered DNA damage, as shown by γ‐H2AV accumulation in *βgal* KD cells (Figure 3d). Importantly, *NELF*‐*A* KD cells have ~five‐fold and ~two‐fold (*p* = 0.02) lower γ‐H2AV level as compared to *βgal* and *NELF*‐*E* KD cells, respectively (Figure 3d). This indicates that *NELF*‐A KD confers more robust protection against DNA damage induced by oxidative stress as compared to depleting other *NELF* subunits. As *Hsp27* gene has been shown to suppress ROS (Wyttenbach et al., 2002), we also examined its expression level in the brains harvested from flies where either *NELF*‐*A* or *NELF*‐*E* gene has been KD by RNAi (Figures 1e and S1G). Similar to S2 cells, KD of *NELF* genes led to elevated *Hsp27* expression as compared to their control siblings, with significantly higher expression observed in *NELF*‐*A* KD brains (Figure 3e). The higher expression of *Hsp* genes, coupled with more enhanced resistance against oxidative stress upon *NELF*‐*A* KD as compared to *NELF*‐*E* KD, may explain why only perturbation of *NELF*‐*A* can extend animal lifespan (Figure 1c,e).

### 2.4 Reduced DNA damage and protein aggregation in aged NelfA*/+ flies

To gain mechanistic insight into the extended healthspan, we examined several markers of aging in the head/brain tissues harvested from NelfA*/+ flies and their control siblings. Consistent with the notion that *Drosophila* undergoes age‐dependent accumulation of DNA damage, γ‐H2Av level in old D50 *w^1118^* male fly heads is significantly higher than young D10 flies (Figure 3f). In contrast, γ‐H2Av level in the heads of D50 NelfA*/+ flies was drastically lower than their control siblings and aged‐matched *w^1118^* flies (Figure 3f).

ROS accumulation can disrupt protein homeostasis to cause protein aggregation during aging (Reichmann et al., 2018). Given their roles in mediating proteostasis (Richter et al., 2010), we asked if elevated HSPs observed in NelfA*/+ fly heads may regulate the level of protein aggregation. In *Drosophila* brains, Ref(2)P marks ubiquitinated protein bodies for autophagic degradation such that the accumulation of insoluble Ref(2)P fraction denotes the formation of protein aggregates (Bartlett et al., 2011; Nezis et al., 2008). Although Ref(2)P protein in the total head lysates was relatively similar across biological replicates, the level of insoluble Ref(2)P fraction is significantly lower in NelfA*/+ fly heads as compared to their control siblings (Figures 3g and S3G). Similarly, the level of insoluble Ref(2)P protein detected in the *NELF*‐*A* KD brains is also significantly lower than their control siblings (EtOH‐ versus RU486‐treated *ElavGal4*. Switch/*NELFA*‐RNAi flies; Figure S3H,I). These results suggest that elevated level of HSPs in NelfA*/+ flies may attenuate DNA damage and protein aggregation during aging.

### 2.5 Lower ROS level is linked to enhanced stress resistance in NelfA*/+ flies

**FIGURE 4:**
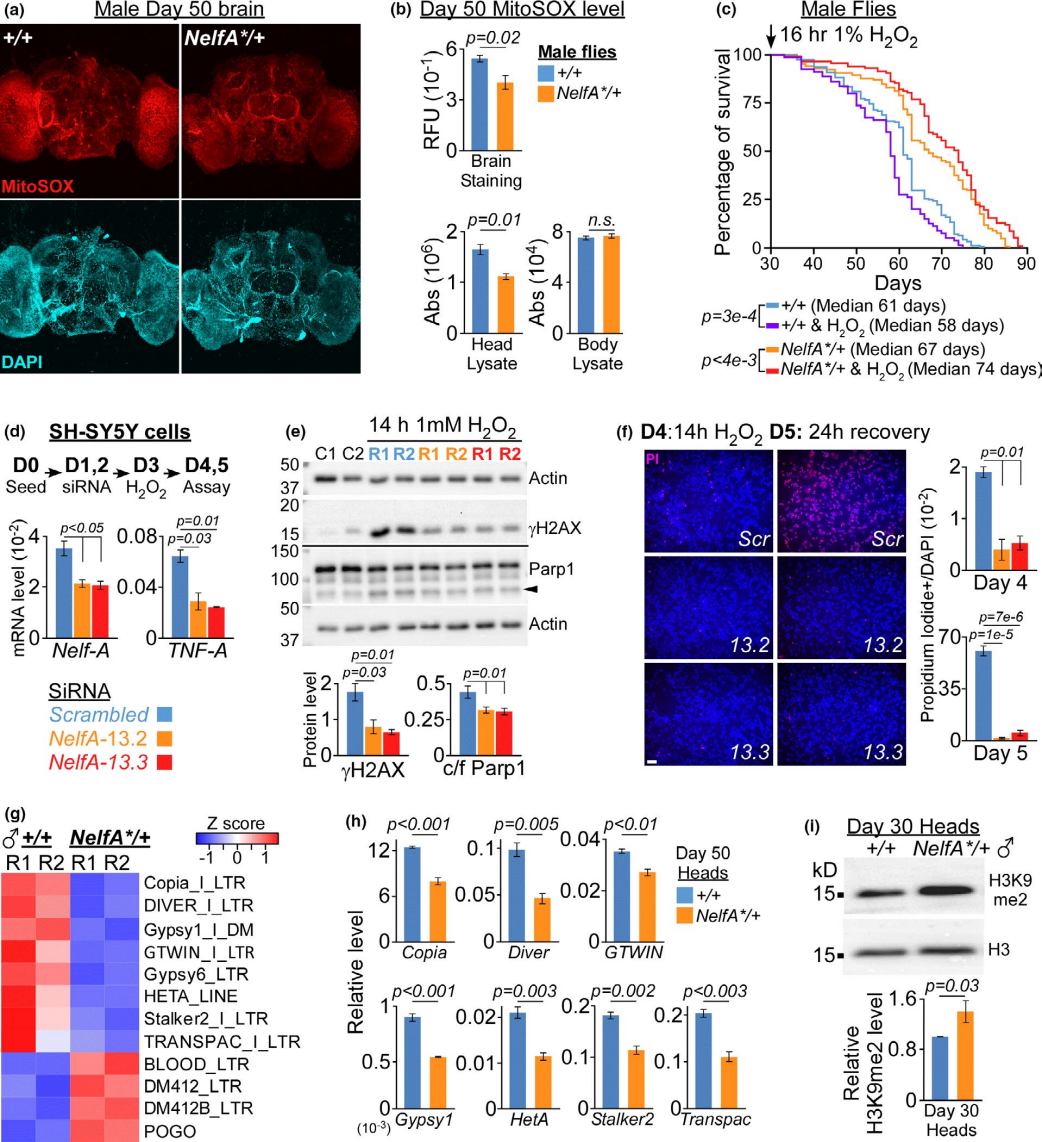
*NELF*‐*A* depletion enhances stress resistance in fly and human cells. (a) Representative confocal images of MitoSOX staining in the brains from D50 male NelfA*/+ flies and their control siblings. (b) Relative fluorescence units (RFU) of MitoSOX staining in the brains normalized to DAPI were presented as mean ± SEM, *n* = 4 (Top). Absorbance (Abs) of MitoSOX signals from the head/body lysates normalized to the protein concentration was presented as mean ± SEM, *n* = 3 (Bottom). Unpaired two‐tailed *t* test. (c) Lifespan of male NelfA*/+ flies and their control siblings that were untreated or exposed to 16 h of 1% H_2_O_2_ at Day 30. *n* = 100–120 flies per group. Log‐rank test. (D) *NELF*‐*A* gene was KD by siRNA on D1 and D2 followed by 14 h of 1 mM H_2_O_2_ treatment on D3. Cells were analyzed on D4 or D5 following 24 h recovery (Top). mRNA expression of different genes on D4. Data presented as mean ± SD, *n* = 2, two‐tailed *t* test (Bottom). (e) Immunoblot of cells lysates harvested on D4. Arrowhead indicates cleaved Parp1 (Top). Quantification of γH2AX and cleaved/full‐length (c/f) Parp1. Data presented as mean ± SD, *n* = 3, two‐tailed *t* test (Bottom). (f) Representative images of PI/DAPI staining of cells on D4 (*n* = 2) and D5 (*n* = 3) following H_2_O_2_ treatment (Left). Scale bar is 50 µM. Quantification with data presented as mean ±SD, two‐tailed t test (Right). (g) Heatmap showing retrotransposons that are differentially expressed in NelfA*/+ and their control sibling heads. R: Biological replicate. (h) RT‐PCR validation of retrotransposons in the heads harvested from D50 NelfA*/+ flies and their control siblings. Data presented as mean ± SEM, *n* = 3, unpaired two‐tailed *t* test. (i) Immunoblot of H3K9me2 in the heads of NelfA*/+ flies and their control siblings (Top). Quantification of H3K9me2 level normalized to H3 and presented as mean ± SEM, *n* = 3, unpaired two‐tailed *t* test (Bottom)

To determine if age‐dependent ROS accumulation is substantially different between NelfA*/+ flies and their control siblings, D50 brains were stained with MitoSOX^TM^ Red reagent, a dye that produces red fluorescence when oxidized by superoxide in the mitochondria. Imaging showed that the normalized MitoSOX signal in NelfA*/+ brains is significantly lower than their control siblings (Figures 4a,b, and S4A). Similarly, MitoSOX fluorescent signal is also reduced in the head, but not in the body lysates prepared from NelfA*/+ flies (Figure 4b), suggesting that NELF‐A may modulate ROS level specifically in the head/brain tissues. To determine the effect of knocking down *NELF*‐*A* gene in neuronal populations, MitoSOX signal was measured in the head and body lysates prepared from male *ElavGal4*. Switch/*NELFA*‐*RNAi* flies. Interestingly, pan‐neuronal KD of *NELF*‐*A* reduces the level of ROS in the head, but not in the body lysates (Figure S4B). This suggests that manipulating the level of NELF‐A in the brains, presumably through HSPs, is sufficient to inhibit ROS accumulation in the heads during aging. To further test if the lower ROS level translates into better protection against environmental stress, D30 NelfA*/+ flies and their control siblings were exposed to 16 hours of 1% H_2_O_2_ treatment. Surprisingly, male NelfA*/+ flies lived longer (~10% higher than untreated NelfA*/+) after H_2_O_2_ treatment whereas the mean lifespan of control siblings was reduced (~5% lower than untreated control siblings; Figure 4c). In contrast, female flies appeared to tolerate this short‐term H_2_O_2_ treatment (Figure S4C). Taken together, halving the level of NELF‐A not only lowers the accumulation of ROS, DNA damage, and protein aggregation in the head/brain tissues, it also enhances resistance against environmental stress in male NelfA*/+ flies.

### 2.6 *NELF*‐*A* depletion enhances stress resistance in human SH‐SY5Y cells

Regulated release of paused RNAPII is crucial for the activation of pro‐survival transcriptional programs upon genotoxic stress in human cells (Bugai et al., 2019), suggesting that *NELF*‐*A*‐mediated stress responses might be highly conserved across species. Consistent with this notion, genes with highly paused RNAPII promoters are associated with DNA damage pathways in human SH‐SY5Y cells (Figure S4D, Table S2). It is plausible that depleting *NELF*‐*A* gene can facilitate productive transcription of these genes to enhance cellular stress responses like in *Drosophila*. To test this, *NELF*‐*A* gene in SH‐SY5Y cells was KD for two consecutive days (D1 and D2) by independent siRNA molecules (*NelfA*‐13.2 and *NelfA*‐13.3) before 14 h of 1 mM H_2_O_2_ treatment (D3). Cells were then harvested immediately after H_2_O_2_ treatment (D4) or allowed additional 24 h of recovery (D5; Figure 4d). Compared to scrambled (*Scr*) siRNA KD, halving the level of *NELF*‐*A* expression in SH‐SY5Y cells led to lower production of pro‐inflammatory cytokine *TNF*‐*A* (Figure 4d), reduced DNA damage marker γ‐H2AX, and apoptotic cleaved Parp1 molecules after 14 h of H_2_O_2_ treatment (Figure 4e). This indicates that depleting *NELF*‐*A* in SH‐SY5Y cells confers better protection against H_2_O_2_‐induced oxidative stress. Consistent with this notion, propidium iodide (PI) staining on D4 or after additional day of recovery (D5) showed that *Scr* KD cells have significantly higher incidence of apoptosis than *NELF*‐*A* KD cells (Figure 4f). Taken together, our results from *Drosophila* S2 and human SH‐SY5Y cells demonstrate that reducing the level of *NELF*‐*A* is sufficient to prevent DNA damage and enhance resistance against oxidative stress.

### 2.7 H3K9me2‐mediated repression of retrotransposons in NelfA*/+ flies

Accumulation of ROS and DNA damage can impact genome integrity through the activation of transposable elements (TEs; Stoycheva et al., 2010). Using TEtranscripts software, which includes TE‐associated ambiguously mapped reads in the differential expression analysis (supplementary experimental procedure), we saw significant changes in the level of specific classes of retrotransposons between D50 male NelfA*/+ flies and their control siblings (Figure 4g). Their differences were further validated by RT‐PCR with additional biological replicates (Figure 4h). The repression of *Gypsy* retroelements in NelfA*/+ flies might partially explain their improved climbing ability (Figure 1d) since age‐dependent locomotor decline has been attributed to *Gypsy* activation in the neuronal populations (Li et al., 2013).

**FIGURE 5:**
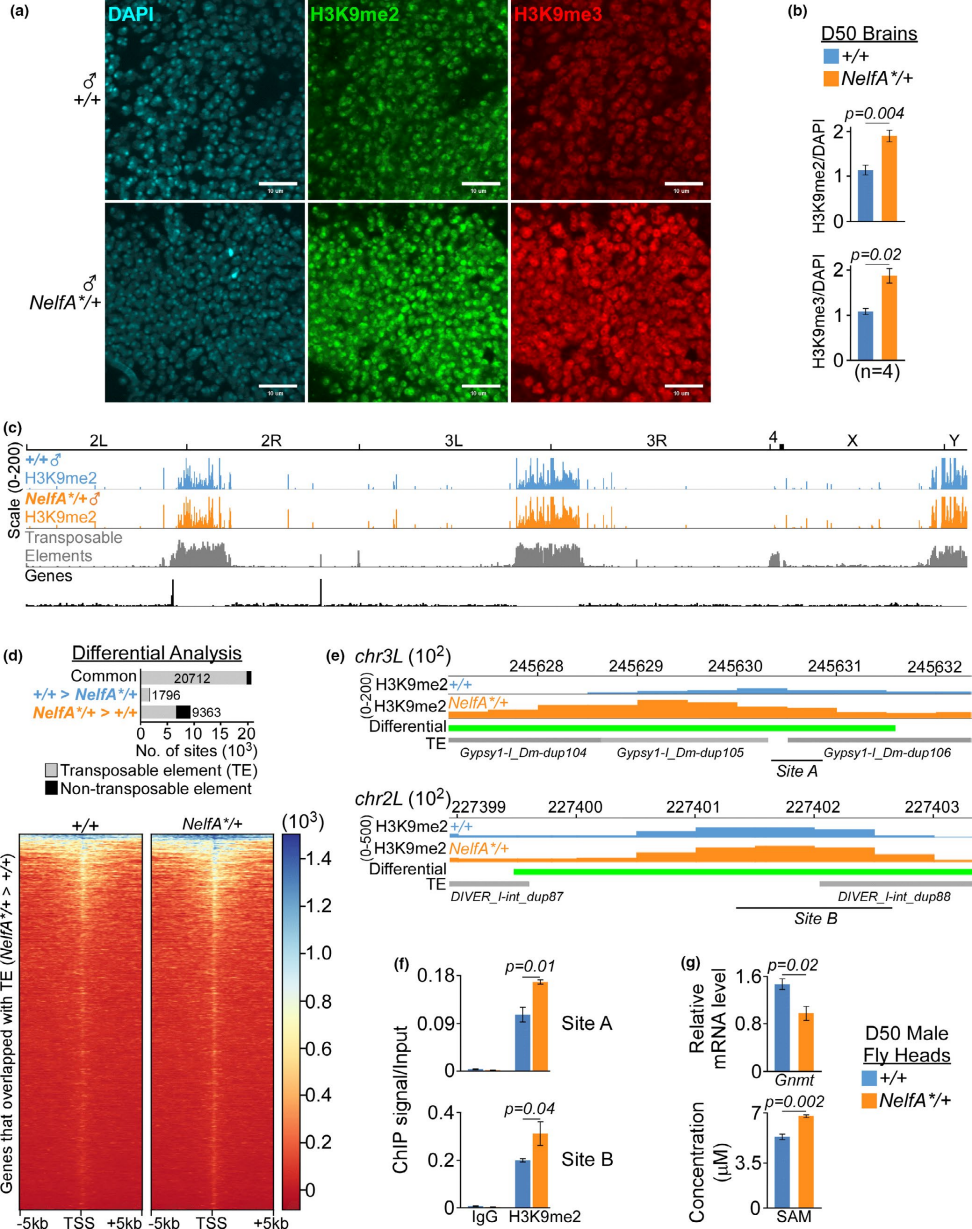
Repression of specific TEs by H3K9me2 in aging NelfA*/+ fly heads. (a) Confocal images of H3K9me2/3 staining in the brains of D50 male NelfA*/+ flies and their control siblings. Scale bar is 10 µM. (b) Quantification of H3K9me2/3 staining in D50 brains from NelfA*/+ flies and their control siblings. H3K9me2/3 normalized to DAPI was presented as mean ± SEM, *n* = 4, paired two‐tailed *t* test. (c) Integrated genomic view (IGV) of H3K9me2 MACS peaks and TEs in the D50 heads of NelfA*/+ flies and their control siblings. (d) Top: Numbers of differential H3K9me2 MACS peaks between NelfA*/+ flies and their control siblings that overlapped with TEs. Bottom: Heatmap of differential H3K9me2 peaks that are more enriched in NelfA*/+ fly heads and overlap with TEs. (e) IGV of H3K9me2 signals from NelfA*/+ flies (orange) and their control siblings (blue), differential peaks called by MACS bdgdiff (green), TE insertion (gray), and qPCR sites. (f) ChIP‐qPCR validation of H3K9me2 signal at site A and B. Data presented as mean ± SD, *n* = 2, unpaired one‐tailed *t* test. (g) Quantification of *Gnmt* and SAM level in the heads of D50 NelfA*/+ flies and their control siblings. Data presented as mean ± SEM, *n* = 3, unpaired two‐tailed *t* test

Loss of H3K9me2/3‐heterochromatin during aging has been linked to increased TEs activation in older flies (Chen et al., 2016; Wood et al., 2016). Since heterochromatin can be regulated by transcription activities and oxidative DNA damage (Frost et al., 2016), we examined the level of H3K9me2/3 in male NelfA*/+ flies and their control siblings. Immunoblot of head lysates showed that the level of H3K9me2 in NelfA*/+ flies is about 1.4‐fold higher than their control siblings (Figure 4i). Similarly, imaging of D50 NelfA*/+ fly brains also showed higher level of H3K9me2 and H3K9me3 staining than their control siblings (Figures 5a,b, and S5A). To understand how TEs are regulated by genome‐wide H3K9me2 distribution, ChIP‐seq was performed in heads of D50 male NelfA*/+ flies and their control siblings (Figure S5B). In line with its regulatory roles in the heterochromatin, H3K9me2 peaks were enriched across the pericentromeric regions where majority of the retrotransposons reside (Figure 5c). Using adjusted *p*‐value <0.05 as cut‐off, MACS bdgdiff identified approximately 20,712 non‐differential, common H3K9me2 peaks; 9363 peaks with higher H3K9me2 level in NelfA*/+ flies; and 1,796 peaks with higher H3K9me2 level in the control siblings (Figure 5d, Table S3). More than 70% (6700/9363) of the differential H3K9me2 peaks in NelfA*/+ flies overlap with known TEs; and are located within the promoter, intronic, and distal intergenic regions (Figures 5d and S5C). The expression of *Gypsy* and *Diver* in NelfA*/+ flies is significantly lower than their control siblings. Of the TEs bound by H3K9me2, about 25% of the *Gypsy* and *Diver* TEs contain differential H3K9me2 peaks that are more enriched in NelfA*/+ flies (Figure S5D, Table S3). The differential enrichment of H3K9me2 across specific *Diver2* and *Gypsy1* loci was furthered validated by ChIP‐qPCR (Figure 5f). In contrast, there is lower percentage of differential H3K9me2 peaks at *DMRC1A*, whose expression is highly similar between NelfA*/+ flies and their control siblings (Figure S5D). These results suggest that lower NELF‐A level may promote the maintenance of H3K9me2‐marked heterochromatin regions, which in turn represses specific TEs like *Gypsy* and *DIVER* in the NelfA*/+ flies.

### 2.8 GNMT may promote H3K9me2 level through SAM in NelfA*/+ fly heads

To understand what contributes to the increased H3K9me2/3 level in old NelfA*/+ flies, we examined the expression pattern of genes that regulate H3K9me2/3 modifications directly or via carbon metabolism (Table S1). Unlike *Su(var)3*–*9*, *dKDM4A*,, and *dKDM4B* genes, the expression of glycine N‐methyltransferase (*Gnmt*) was significantly downregulated in NelfA*/+ flies (Figure 5g). GNMT is a metabolic enzyme that regulates the ratio of S‐adenosylmethionine (SAM) to S‐adenosylhomocysteine (SAH; Luka et al., 2009). Histone methyltransferase (HMT) utilizes SAM as the methyl donor to methylate histone residues, in the process releasing SAH which may inhibit HMT *via* negative feedback mechanism. In *w^1118^* fly heads, there is significant increase in *Gnmt* expression and concomitant drop in SAM concentration during aging (Figure S5G). The lower expression of *Gnmt*, which coincides with increased SAM concentration, might contribute to the maintenance of H3K9me2‐enriched heterochromatin in the heads/brains of old NelfA*/+ flies as compared to their control siblings.

## 3 DISCUSSION

Through functional analyses and epigenome profiling, we showed that the level of NELF‐A in the head tissues plays an important role in regulating *Drosophila* healthspan. As RNAPII pausing is the rate‐limiting step in the transcription of many *Hsp* genes (Lis et al., 2000; Wu et al., 2003), lowering the level of NELF‐A in either NelfA*/+ flies or by pan‐neuronal *NELF*‐*A* KD might facilitate the release of paused RNAPII and increase its productive elongation of these genes (Figure 3a,b). Consistent with their roles in extending lifespan (Biteau et al., 2010; Morrow et al., 2004; Wang et al., 2004) and suppressing neurotoxicity in fly Huntington's disease model (Warrick et al., 1999), our data indicate that elevated HSPs are likely driving the improved healthspan in *NELF*‐*A* depleted flies (Figure 1). Indeed, the level of systemic inflammation (Figure 2), DNA damage (Figure 3f), protein aggregation (Figure 3g), and ROS (Figure 4) in the brains/heads of NelfA*/+ flies is significantly lower than their control siblings. These molecular signatures also coincide with their enhanced resistance against external oxidative stress during aging (Figure 4c). Moreover, as oxidative DNA damage can lead to chromatin relaxation and heterochromatin loss (Frost et al., 2016), reduced ROS may also promote the maintenance of H3K9me2‐enriched heterochromatin and concomitant repression of TEs in aged NelfA*/+ flies as compared to their control siblings (Figure 5). Although H3 K9 methylation is more favorable with the increased SAM concentration, it remains unclear how *Gnmt* expression may be downregulated in NelfA*/+ flies.

Unexpectedly, animal lifespan is only affected by *NELF*‐*A* perturbation even though depleting other *NELF* subunits was sufficient to induce the expression of *Hsp* genes in S2 cells (Gilchrist et al., 2008). Biochemical and structural studies showed that NELF‐A mobile C‐terminal domain plays a more important role in stabilizing RNAPII pausing complex than NELF‐E subunit (Narita et al., 2003; Vos et al., 2018). Consistent with these findings, there is higher expression of *Hsp* genes in conjunction with reduced DNA damage in *NELF*‐*A* KD cells as compared to *NELF*‐*E* KD cells (Figure 3c‐e). *In vitro* RNA extension assay reveals that NELF‐A, and not NELF‐E, is required for RNAPII pausing (Vos et al., 2018). It is likely that perturbating *NELF*‐*E* or other subunits might not elicit sufficient change in the level of HSPs to influence animal lifespan. Moreover, the expression of *NELF*‐*A* gene in fly heads is ~7–10‐fold higher than *NELF*‐*E* and *NELF*‐*B* respectively (Figure S2F), suggesting that *NELF*‐*A* may play a more dominant role in gene regulation than other *NELF* subunits in the head tissues.

In aging flies, elevated Rel level in the fat bodies and brains triggers systemic inflammation to cause gut hyperplasia (Chen et al., 2014) and neurological decline, respectively (Kounatidis et al., 2017). Interestingly, NELF depletion has been shown to attenuate immune responses *via* distinct mechanisms in different model systems. In S2 cells and *Drosophila* larvae, *NELF* KD downregulates the basal expression of many components of the immune signaling pathways (including Rel), thereby reducing the activation of *AMP* genes (Gilchrist et al., 2012). In mouse macrophage, knocking out *NELF* increases the expression of key anti‐inflammatory cytokine IL‐10 through AP‐1‐dependent transcriptional circuit (Yu et al., 2020). Despite having similar level of Rel, the brains/heads of D50 NelfA*/+ flies has significantly lower *AMPs* expression than their control siblings. This is indicative of less systemic inflammation, a likely consequence of enhanced HSP‐mediated protection. The potential pro‐longevity effects of other neuropeptides and signaling pathways in NelfA*/+ flies will warrant further studies (Figure 2b).

The pro‐longevity role of NELF‐A appears to be restricted to the brains since depleting NELF‐A in the neuronal populations, but not in fat bodies, affects the animal healthspan (Figure S1). Similarly, ROS level is significantly reduced only in the brain/head tissues of NelfA*/+ flies as compared to their control siblings (Figure 4b). In developing mouse cortex, epigenetic profiling of neural progenitor cells (NPC) and differentiated neurons revealed that the promoters of many DNA damage response and repair genes have highly paused RNAPII (Liu et al., 2017). Moreover, GO analysis showed that these genes are associated with molecular functions like NF‐κB and HSP binding (Figure S5D, Table S2), suggesting the release of paused RNAPII may be the rate‐limiting step in their transcription. In human cells, activation of P‐TEFb by RNA‐binding motif protein 7 is necessary to release paused RNAPII for productive transcription of DNA damage response genes upon genotoxic stress (Bugai et al., 2019). Consistent with these findings, *NELF*‐*A* KD in human SH‐SY5Y cells led to better protection against H_2_O_2_‐induced DNA damage and apoptosis than *Scr* KD cells (Figure 4d,f). These results suggest that regulation of stress‐responsive genes by NELF‐A mediated RNAPII pausing is likely to be conserved across different species.

Although it remains to be seen if *NELF*‐*A* affects lifespan in mammals, several lines of evidence indicate that lowering the level of NELF may have wider implications in regulating human health and disease state. NELF‐A is a prognostic marker of liver cancer such that higher level of protein expression correlates with unfavorable survival probability in patients (proteinatlas.org). Conversely, knocking down *NELF*‐*B* gene in hepatocellular carcinoma cells is sufficient to reduce cellular proliferation and migration (El Zeneini et al., 2017). In mouse, *microRNA*‐*133 *has been demonstrated to inhibit cardiac hypertrophy through repressing the translation of *RhoA*, *Cdc42*, and *NELF*‐*A* mRNA (Care et al., 2007). Conversely, overexpression of NELF‐A in cardiomyocytes can induce hypertrophic gene program (Care et al., 2007). Further studies on RNAPII pausing in mammalian systems under different contexts might provide insights into its roles in regulating stress responses and aging transcriptome. Investigation of direct NELF targets would complement these studies and potentially lead to the discovery of pro‐longevity or specific therapeutic strategies.

### 3.1 Experimental procedures

Additional experimental procedures are described in supporting information (SI).

### 3.2 Fly strains

The following lines were from the Bloomington Stock Center (BL): *Nelf*‐*A^KG09483^* (BL#15189), *Nelf*‐*A* RNAi HMS00686 (BL#32897), *Nelf*‐*B* RNAi HMS00165 (BL#34847), *Nelf*‐*D* RNAi HMS00283 (BL#42931), *Nelf*‐*E* RNAi *P{UAS*‐*Nelf*‐*E*.*IR}17A10* (BL#6788). *Hsp70*‐*Gal4* line was from Dr Yu CAI. GeneSwitch (GS) tissue‐specific *Gal4* drivers used: pan‐neuronal *elav*‐*Gal4*::*GS* (BL#43642), abdomen fat‐body *106*‐*Gal4*::*GS* (BL#8151) and head fat‐body *bun*‐*Gal4*::*GS* (BL#8527).

### 3.3 Transgenic flies

PCR amplified full‐length *NELF*‐*A* (SD07139, DGRC) and *NELF*‐*E* (RE14181, DGRC) cDNAs were cloned into pENTR/D‐TOPO and pAWM vectors with Gateway kit (Invitrogen). *NELF* cDNAs with c‐terminal myc‐tag were PCR amplified from pAWM vector, cut with *BamHI*/*XbaI*, and cloned into *BglII*/*Xbal* sites on *pUAST* vector. Constructs were sequenced and sent to Bestgene Inc to make transgenic fly. Homozygous lines (*w^1118^* background) are denoted as *UAS*‐*NelfA*‐*6myc* and *UAS*‐*Nelf*‐*E*‐*6myc*.

### 3.4 Ectopic expression or tissue‐specific depletion of different NELF subunits

*Hsp70*‐*Gal4* females were backcrossed to *w^1118^* males for >10 generations to obtain *Hsp70*‐*Gal4*/+ flies. Backcrossed *Hsp70*‐*Gal4*/+ virgin females were mated with homozygous *UAS*‐*NelfA*‐*6myc* or *UAS*‐*Nelf*‐*E*‐*6myc* males. F1 offspring were cultured in 30°C to induce ectopic *NELF* expression.

The *UAS*‐*RNAi* males were mated to *GeneSwitch* drivers. F1 offspring were cultured in media containing either ethanol or 120 mg/ml of mifepristone (RU486, Sigma).

### 3.5 Lifespan assay

*Nelf*‐*A^KG09483^*/*Tm3* virgin females were backcrossed to *w^1118^* males for >10 generations to obtain *Nelf*‐*A^KG09483^*/+ mutants (NelfA*/+) and control siblings (+/+) with similar genetic background. Twenty flies were housed in a vial (diameter 2.5 cm, height 9.5 cm) with 5 ml of standard fly media. Food was changed every two days with daily recording. Data were plotted with Prism GraphPad and statistical significance calculated by log‐rank Mantel‐Cox's test. There were at least 100 flies per aging assay.

### 3.6 Negative geotaxis (climbing) assay

About 10–15 age‐matched flies were transferred to fresh food a day before assay. On the day of assay, flies were transferred without CO_2_ anesthesia into glass cylinder (height 22 cm, diameter 2.5 cm, capacity 50 ml, AS ONE) capped with cotton plug. The flies were tapped to the bottom and distance ascended in 6 s (D20, 30) or 10 s (D50) was captured by digital camera. This was repeated to obtain sizeable number for each genotype. Data were plotted with Prism GraphPad and statistical significance calculated by Mann–Whitney test.

### 3.7 Oxidative stress test and ROS measurement

For short‐term stress, Day 30 (D30) flies were transferred to 1% agar containing 1% sucrose, 1% nipagin, 1% H_2_O_2_ for 16 h and returned to regular media. To image brain ROS level, D50 fly brains dissected in cold Hank's Balance Salt solution (HBSS) were incubated with 5 μM MitoSOX (Invitrogen, M36008) and Hoechst for 30 min at room temperature. After three rounds of cold PBS wash, brains were mounted in VECTASHIELD (Vector Labs). Z‐stack images were taken with confocal microscope (FV3000, 20X objective) and the total fluorescence intensity was quantified with ImageJ. Relative cells fluorescence unit (CFU) was obtained by dividing MitoSOX signal over Hoechst staining. To quantify ROS level in the lysates, 10 heads or bodies dissected from D50 flies were homogenized in cold PBS with protease inhibitors cocktail (Roche) and sonicated (30 s ON/OFF, high settings, 5 cycles, Bioruptor UCD‐200TO). 10 μl of diluted (1:5) lysates were incubated in 300 μl of 5 μM MitoSOX for 10 min at room temperature. Fluorescence was measured by luminescence spectrometer (Tecan Spark Multimode Reader, SCIMED) at 510 nm excitation and 580 nm emission. The absorbance was normalized to the protein concentration (Bradford assay, Bio‐Rad).

### 3.8 Immunoblot and antibodies

For total lysates, 10 fly heads were homogenized in 100 μl of 1 x Laemmli buffer and boiled for 10 min. To separate soluble from insoluble fraction (Bartlett et al., 2011), 10 fly brains/heads were homogenized in ice‐cold 1% Triton‐X 100 in PBS with protease inhibitors cocktail (Roche). About 10% volume was kept as total fraction while the rest centrifuged at 14,000 g for 10 min at 4°C. The supernatant collected is the soluble fraction while the pellet was resuspended in SDS extraction buffer (50 mm Tris, pH 7.5, 2% SDS, protease inhibitors cocktail) and sonicated. Following 10 minutes of 14,000 g centrifugation at room temperature, the supernatant from the SDS extraction buffer was collected as the insoluble fraction. Antibodies used are: NELF‐A (in‐house, 1:500), Myc (A14, Santa Cruz, 1:3000), Ref(2)P (ab178440, Abcam, 1:500), γ‐H2Av (UNC93‐5.2.1, DSHB, 1:500), *β*‐tubulin (E7, DSHB, 1:3000), lamin‐Dm0 (67.10‐s, DSHB, 1:3000), H3K9me2 (ab1220, Abcam, 1:1000), and histone H3 (ab1791, Abcam, 1:5000).

### 3.9 RNA isolation, RNA‐seq, and data processing

Total RNA was isolated from 20 x D50 fly heads using RNAzol®RT (Sigma) protocol and sequenced by BGI (Hong Kong). RNA‐seq dataset was processed and analyzed according to previous methods detailed in SI.

### 3.10 Chromatin Immunoprecipitation (ChIP) and sequencing

ChIP and library amplification were performed according to previous protocol detailed in SI. For each biological replicate, 100 fly heads and 5 μg of antibody (H3K9me2, ab1220 and RNAPII, CTD4H8) were used.

### 3.11 S2 cells culture, dsRNA KD, and H_2_O_2_ treatment

*NELF*‐*A* and *NELF*‐*E* dsRNA was generated with Ambion MEGAscript T7 kit using PCR template. On Day 0 (D0), 0.5e^6^ of S2 cells were seeded in 0.5 ml of serum‐free medium (SFM, Gibco™, 21720024) in a 24‐well plate. Cells were transfected with 4 µg of dsRNA (βgal, *NELF*‐*A*, and *NELF*‐*E*) with 4 µl of Cellfectin™ II (ThermoFisher, 10362100) for 2 h. SFM was replaced with 0.5 mL of full media. After 24 h (D1), second round of transfection was performed with 8 µg of dsRNA and 8 µl of Cellfectin. On D2, cells were split evenly into two wells and incubated with either 1 or 5 mM of H_2_O_2_ for 16 h. On D3, cells were washed twice with PBS and harvested for analyses.

### 3.12 siRNA KD and H_2_O_2_ treatment of SH‐SY5Y cells

About 0.2e^6^ cells/well were seeded in a 24‐well plate on Day 0 (D0). Cells were transfected with 40 nM of Dicer‐Substrate Short Interfering RNAs (DsiRNA) using 0.75 µl of Lipofectamine^TM^ 3000 and 1 µl of P3000 per reaction (Invitrogen) on D1. Scrambled control (51‐01‐19‐09) and *NELF*‐*A* (hs. Ri.NELFA.13.2 or hs. Ri.NELFA.13.3). DsiRNA were purchased from IDT. Following 24 h of incubation, cells were replenished with 0.5 ml of fresh media and transfected again with 40 nM of DsiRNA (D2). On the following day, cells were replaced with fresh media containing 1 mM of H_2_O_2_ (D3). Following 14 h of H_2_O_2_ treatment, cells were washed twice with PBS to remove dead floating cells (D4). The attached cells were either harvested for analyses (western/mRNA/staining with propidium iodide and DAPI) or replenished with fresh medium (D4). Following 24 h recovery, cells were stained with PI and DAPI (D5). The antibodies used were phospho‐H2AX (sc‐517348), PARP‐1 (Active Motif 39561), and β‐actin (A2228, Sigma).

### 3.13 Primers list

Primers sequences are listed in Table S4.

## CONFLICT OF INTEREST

None declared.

## AUTHOR CONTRIBUTIONS

ZKN & CTO conceived and designed this study. ZKN & WQL performed the experiments. ZKN analyzed experimental results. ZKN & CTO wrote and reviewed the manuscript.

## Supporting information

Supplementary Material

## Data Availability

RNA‐seq and ChIP‐seq dataset have been deposited in GEO with accession code GSE155940.
