# Erratum to: NELF‐A controls *Drosophila* healthspan by regulating heat‐shock protein‐mediated cellular protection and heterochromatin maintenance

**DOI:** 10.1111/acel.13547

**Published:** 2022-01-17

Zhen‐Kai Ngian, Wei‐Qi Lin, Chin‐Tong Ong, *Aging Cell*, 20, e13348. https://doi.org/10.1111/acel.13348

In the published version of Ngian et al. (2021), the authors noticed that the panel label (f) was incorrectly placed, and label (g) was missing in Figure 3. The corrected version of Figure 3 was shown below.

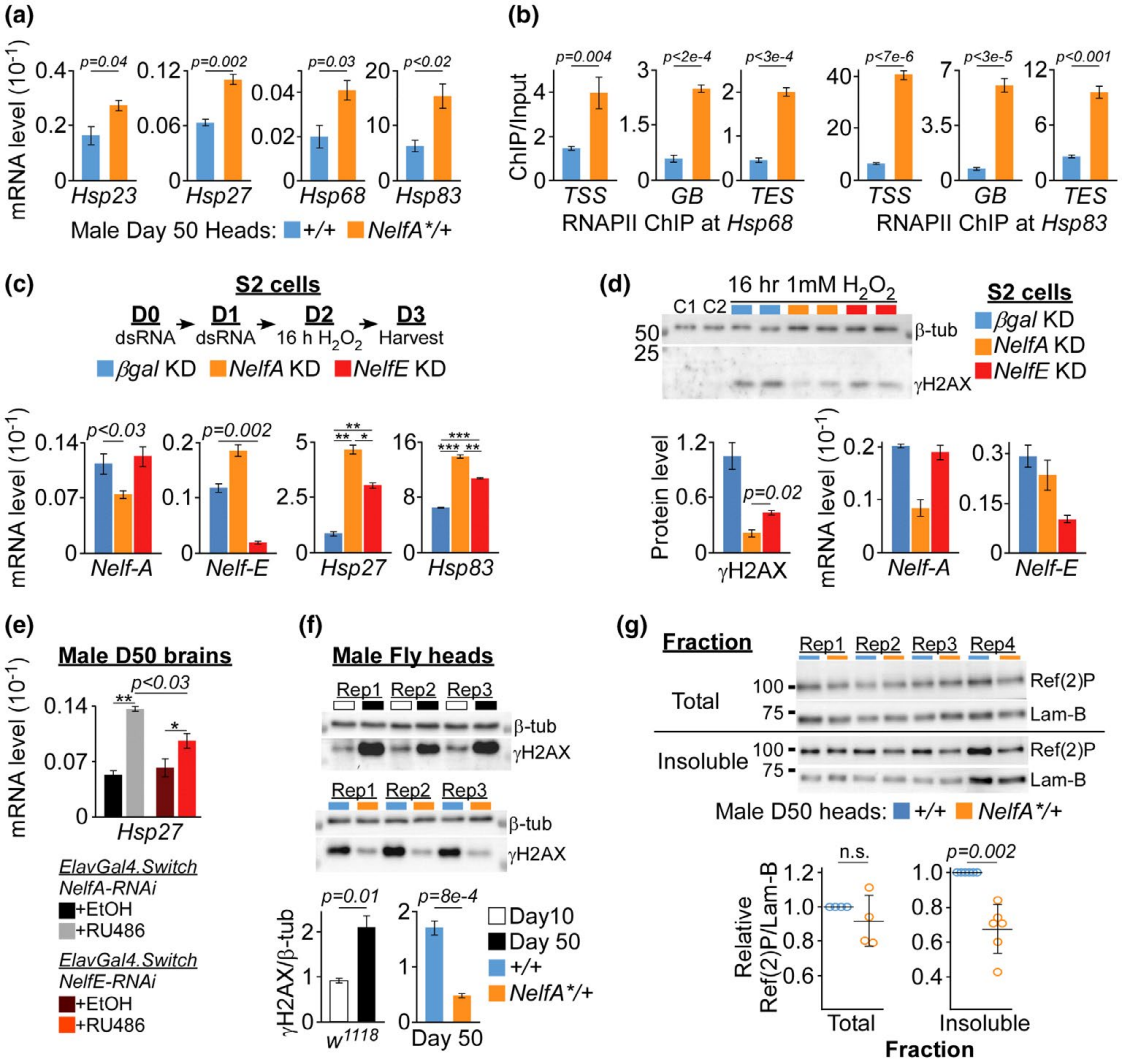

The authors would like to apologize for the inconvenience caused.
