# Supplementary material for: NELF‐A controls Drosophila healthspan by regulating heat‐shock protein‐mediated cellular protection and heterochromatin maintenance
Source: Aging Cell. 2021 Mar 31;20(5):e13348. doi: 10.1111/acel.13348 (PMC8135010; doi:10.1111/acel.13348)
Supplement: Supplementary file 1 — Supplementary Material [file ACEL-20-e13348-s001.pdf]

Appendix S1: Supporting information

NELF-A controls *Drosophila* healthspan by regulating heat-shock protein-mediated cellular protection and heterochromatin maintenance.

Zhen-Kai Ngian[1,2], Wei-Qi Lin[1], Chin-Tong Ong[1,2*]

[1]Temasek Life Sciences Laboratory, National University of Singapore, Singapore 117604, Singapore.

[2]Department of Biological Sciences, National University of Singapore, Singapore 117543, Singapore.

[*]Corresponding author: chintong@tll.org.sg

Supplementary Figures 1-5 and legend

Description of Supplementary Tables

Supplementary experimental procedures

Supplementary references

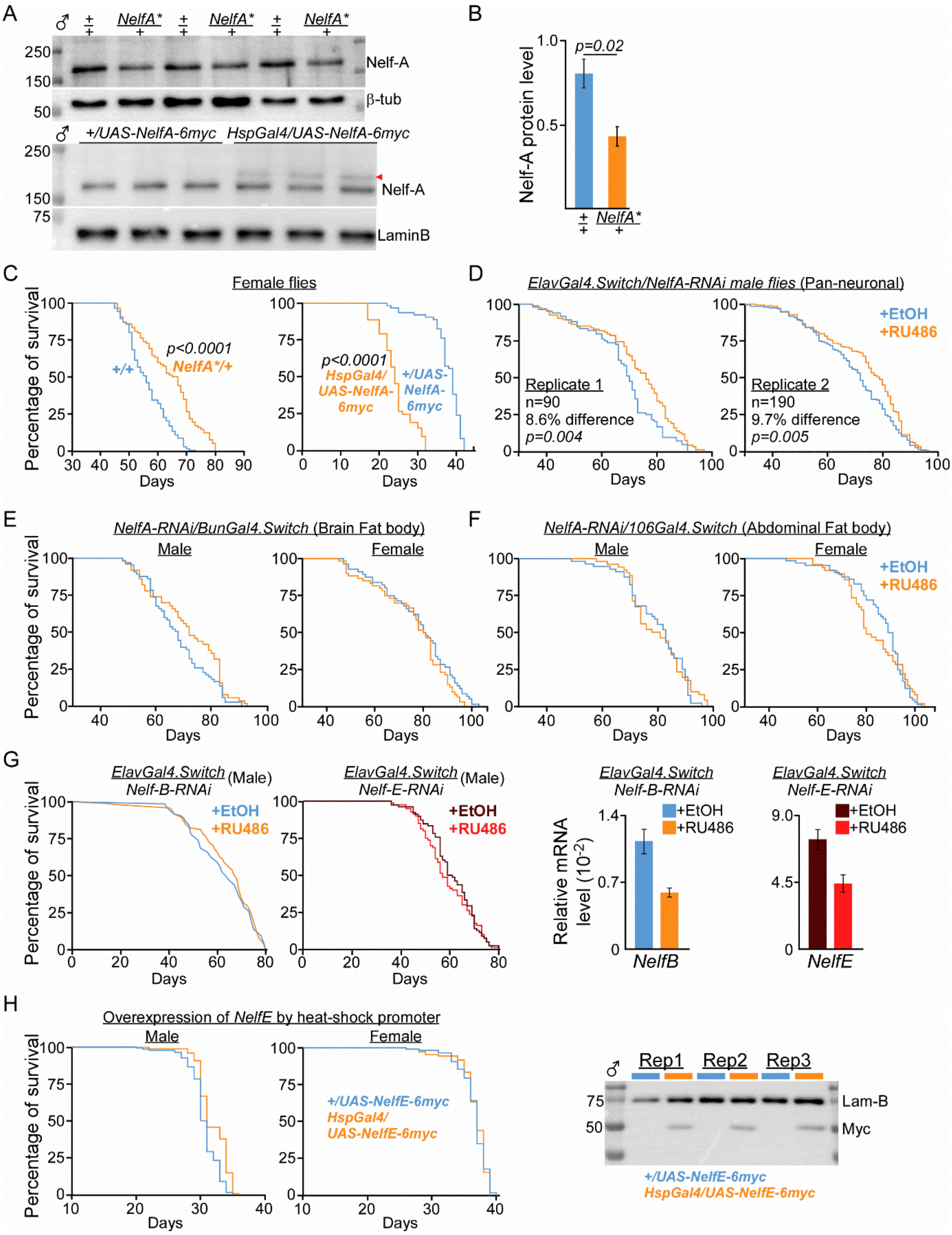
A
NelfA*
Nelf-A
β-tub
+/UAS-NelfA-6myc
HspGal4/UAS-NelfA-6myc
LaminB
B
p=0.02
Nelf-A protein level
C
Female flies
Percentage of survival
p<0.0001
+/+
NelfA*/+
HspGal4/ UAS-NelfA-6myc
+/UAS-NelfA-6myc
Days
D
ElavGal4.Switch/NelfA-RNAi male flies (Pan-neuronal)
Replicate 1
n=90
8.6% difference
p=0.004
Replicate 2
n=190
9.7% difference
p=0.005
+EtOH
+RU486
E
NelfA-RNAi/BunGal4.Switch (Brain Fat body)
Male
Female
F
NelfA-RNAi/106Gal4.Switch (Abdominal Fat body)
G
ElavGal4.Switch (Male)
Nelf-B-RNAi
Nelf-E-RNAi
Relative mRNA level (10⁻²)
NelfB
NelfE
H
Overexpression of NelfE by heat-shock promoter
+/UAS-NelfE-6myc
HspGal4/UAS-NelfE-6myc
Rep1
Rep2
Rep3
Lam-B
Myc

Supplementary Figure 1. Neuronal KD of *NELF-A*, but not other subunits, promotes animal healthspan

(A) Immunoblot of NELF-A protein across multiple biological replicates of *NelfA*/+* flies, *Hsgal4/UAS-NelfA-6myc* lines and their respective control siblings. Each lane contains five male adult fly heads.

(B) Quantification of NELF-A protein in the heads harvested from male *NelfA*/+* flies and their control siblings. Data presented as mean ± SEM, biological replicates = 3, paired two-tailed *t*-test. Each biological replicate has five adults fly heads.

(C) Lifespan assay of female *Nelf-A*/+* flies (+ 19.6%), *Hsgal4/UAS-NelfA-6myc* flies (-32.1%) and their respective control siblings. n = 220-240 from combining two independent aging cohorts. Log-rank test.

(D-H) Perturbation of NELF-A, but not other subunits, affects animal lifespan.

(D) Lifespan assay of EtOH- or RU486-treated *ElavGal4.Switch/NelfA-RNAi* male flies. Log-rank test

(E, F) Lifespan assay of flies where *NELF-A* was KD in either brain or abdominal fat bodies.

(G) Left: Lifespan assay of male flies with pan-neuronal KD of either *NELF-B* or *NELF-E* gene. Right: RT-PCR quantification of *NELF-B* and *NELF-E* genes expression in the brains of the EtOH- and RU486-treated flies.

(H) Left: Lifespan assay of *Hsgal4/UAS-NelfE-6myc* flies and their control siblings. Right: Immunoblot of myc-tag NELF-E protein in *Hsgal4/UAS-NelfE-6myc* flies but not in their control siblings.

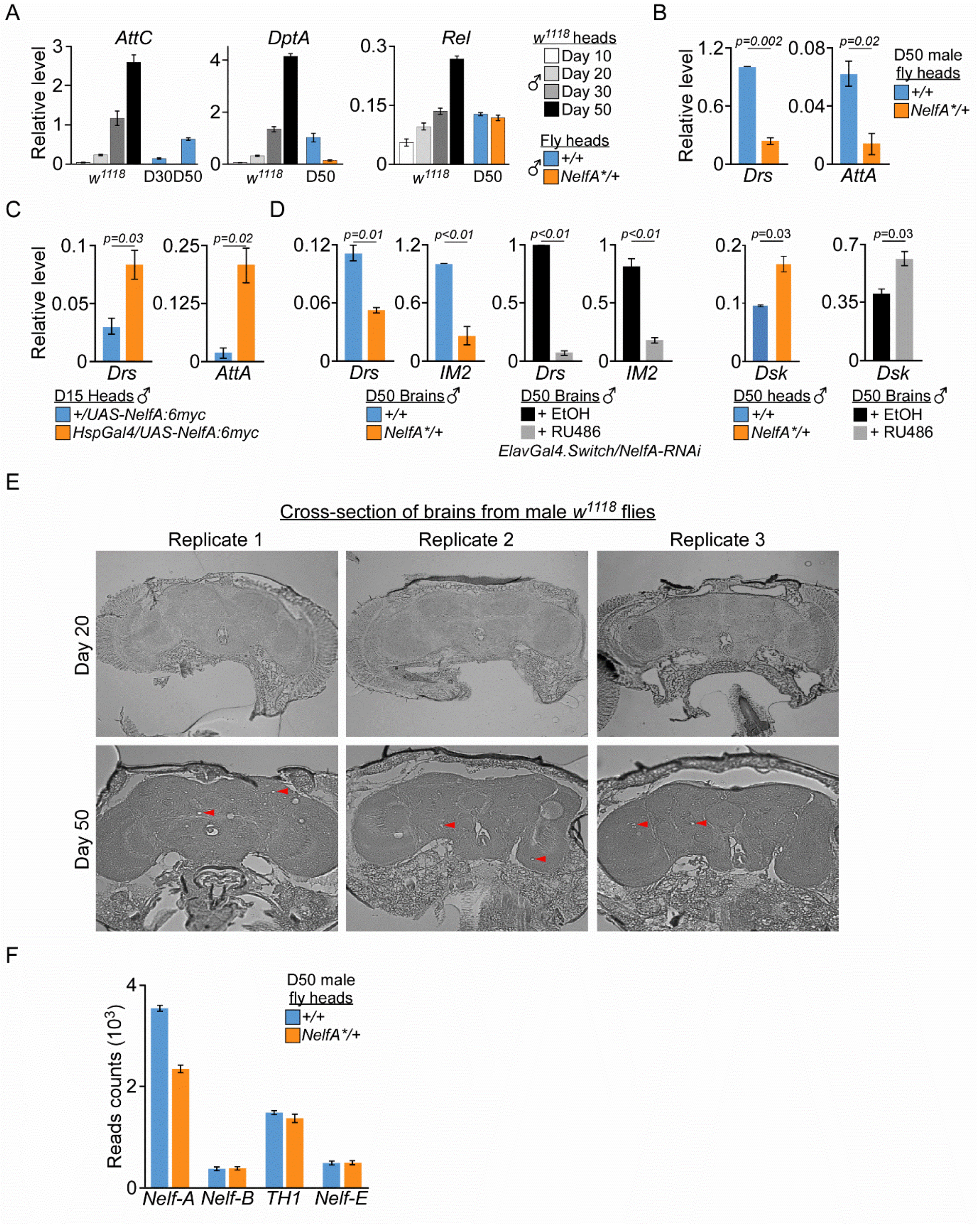

Supplementary Figure 2. Age-dependent increase in immune response genes expression and neurodegeneration are attenuated in *NelfA/+ flies**

(A) Quantification of *AMPs* (*AttC*, *DptA*) and *Rel* expression in the heads of aging w^{1118} flies, Day 50 *NelfA**/+ flies and their control siblings.

(B) Quantification of *AMPs* expression in heads of male *NelfA*/+* flies and their control siblings. Data presented as mean ± SEM, n = 3, unpaired, two-tailed *t*-test.

(C) Quantification of *AMPs* expression in the heads of male *Hsgal4/UAS-NelfA-6myc* flies and their control siblings. Data presented as mean ± SD, n = 2, unpaired, two-tailed *t*-test.

(D) Quantification of *AMPs* expression in the brains isolated from male *NelfA*/+* flies, RU486-treated *ElavGal4*.Switch/*NelfA-RNAi* flies and their respective control siblings. RT-PCR of neuropeptide *Dsk* gene in the fly heads (n = 3) and brains (n =2). Data presented as mean ± SD (n = 2) or SEM (n = 3), unpaired two-tailed *t*-test.

(E) Histological analysis of brain sections prepared from Day 20 and 50 male w^{1118} flies with vacuoles marked by red arrowheads.

(F) Raw RNA-sequencing read counts of different *NELF* subunits in Day 50 heads of male *NelfA*/+* flies and their control siblings.

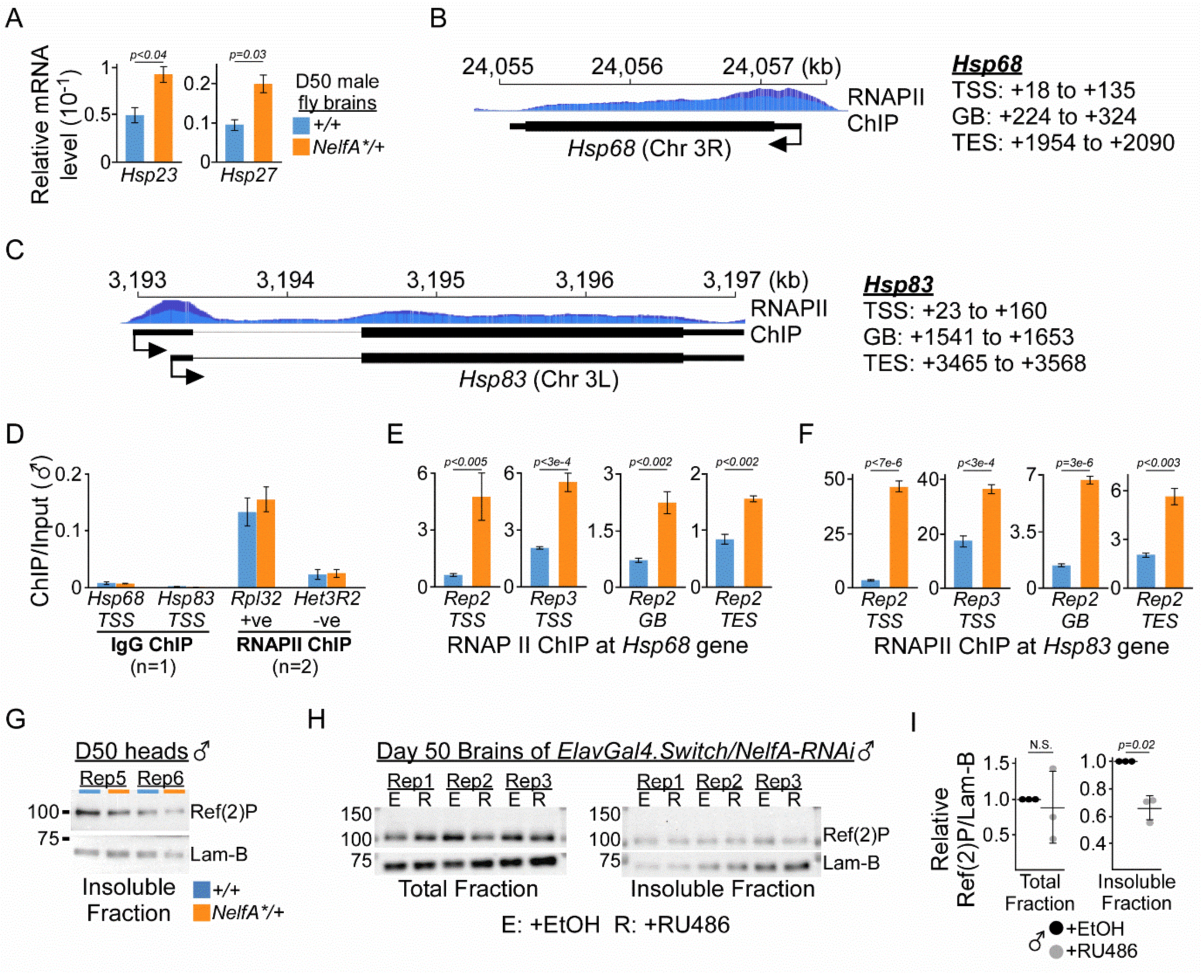

Supplementary Figure 3. Increased RNAPII occupancy on *HSPs* is correlated with higher gene expression and reduced protein aggregation

(A) Quantification of *Hsp23* and *Hsp27* expression in brains isolated from male *NelfA**/+ flies and their control siblings. Data presented as mean ± SD, n = 2.

(B-C) RNAPII ChIP-seq signal over *Hsp68* and *Hsp83* genes in KC167 cells. Genomic coordinates of the primers designed to cover the TSS, GB and TES of the *Hsp* genes.

(D-F) Elevated RNAPII occupancy across *Hsp68* and *Hsp83* genes in male *NELF-A**/+ fly heads as compared to their control siblings. **(D)** IgG- and RNAPII-ChIP enrichment at TSS, the highly expressed *Rpl32* gene and silenced *Het3R2* region. **(E-F)** RNAPII-ChIP enrichment at the TSS, GB, and TES of *Hsp68* and *Hsp83* in heads isolated from male *NelfA**/+ flies and their control siblings. Data was normalized to input and presented as mean ± S.D. from triplicate qPCR reactions. Second biological replicate for Fig.3B.

(G) Immunoblot of insoluble Ref(2)P fraction in the heads of Day 50 male *NELF-A**/+ flies and their control siblings. Biological replicate (Rep) 5 and 6 for Fig.3G.

(H-I) Reduced level of insoluble Ref(2)P fraction in the brains of pan-neuronal *NELF-A* KD male flies. **(H)** Immunoblot of the different fractions prepared from the brains of EtOH- and RU486-treated *ElavGal4.Switch/NELFA-RNAi* flies. **(I)** ImageJ quantification of Ref(2)P in the brains. Data presented as mean ± SD, n = 3. N.S.: not significant. Paired two-tailed *t*-test.

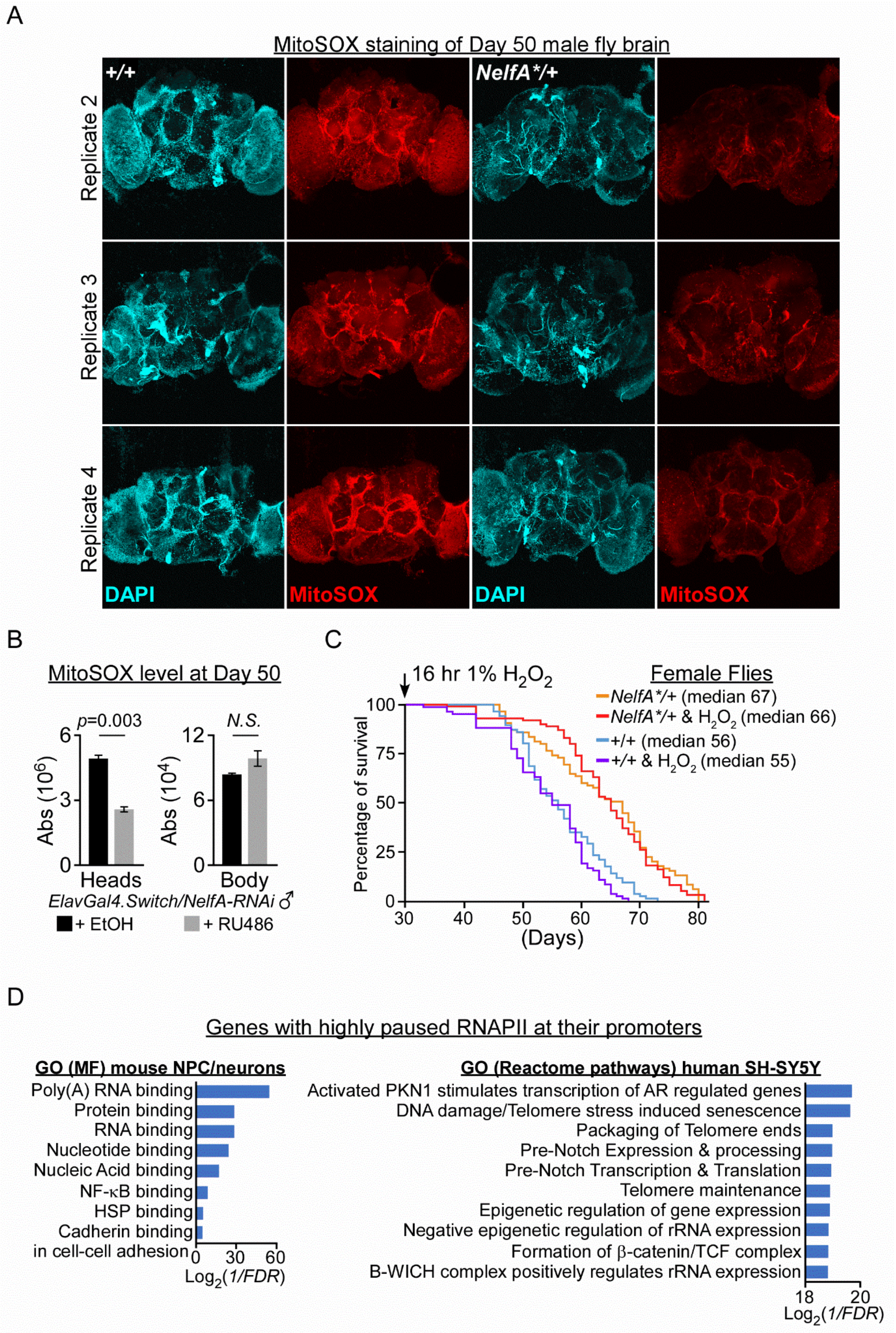

Supplementary Figure 4. Lower level of ROS in *NELF-A* depleted brains

(A) MitoSOX staining of brains isolated from Day 50 male *NelfA*/+* flies and their control siblings. Three additional biological replicates for Fig.4A and 4B.

(B) Absorbance (Abs) measurement of MitoSOX fluorescence intensity in the head and body lysates prepared from EtOH- and RU486-treated *ElavGal4.Switch/NELFA-RNAi* male flies. Data presented as mean ± SEM, n = 3, unpaired two-tailed *t*-test.

(C) Lifespan assay of female *NelfA*/+* flies and their control siblings that were either untreated or exposed to 16 hr of 1% H_2O_2 at Day 30. n = 100 flies per group. Log-rank test.

(D) GO term analysis of genes with highly paused RNAPII promoters in mouse NPCs/neurons (Molecular functions, Left) and human SH-SY5Y cells (Reactome pathways, Right).

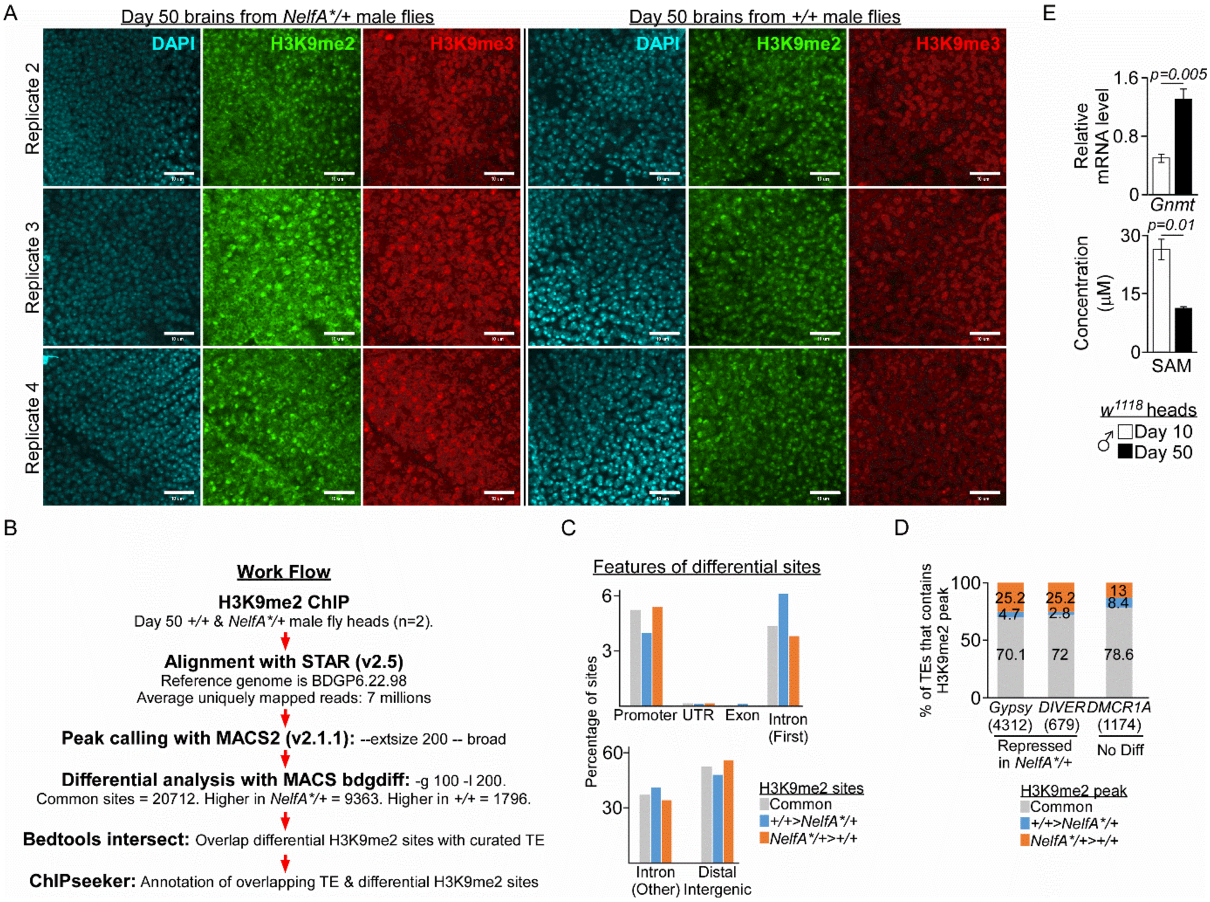

Supplementary Figure 5. Higher concentration of SAM and H3K9me2/3 staining in the brains of *NelfA*/+* flies

(A) Immunostaining of H3K9me2/3 in brains isolated from Day 50 male *NelfA*/+* flies and their control siblings. Three additional biological replicates for Fig.5A and 5B. Scale bar is 10 μM.

(B) Workflow of bioinformatic analysis of H3K9me2 ChIP-seq.

(C) Genomic distribution of the common and differential H3K9me2 sites that overlapped with transposable elements (TEs).

(D) Percentage of different TEs that overlapped with the various types of H3K9me2 peaks. Parentheses indicate the number of TEs sites. The expression of *Gypsy* and *DIVER* is lower in *NelfA*/+* flies, whereas there is no difference (No Diff) in the level of *DMCR1A* between *NelfA*/+* flies and their control siblings.

(E) Quantification of *Gnmt* gene expression (n = 3) and SAM level (n=2) in the heads of Day 10 and Day 50 w^{1118} flies. Data presented as mean ± SEM (n = 3) or SD (n = 2), unpaired two-tailed *t*-test.

<u>Description of Supplementary Tables 1 to 4</u>

Supplementary Table 1

List of differentially expressed genes between heads harvested from Day 50 male *NelfA*/+* flies and their control siblings.

Supplementary Table 2

Gene ontology analysis of differentially expressed genes between heads harvested from Day 50 male *NelfA*/+* flies and their control siblings, the common highly paused genes in mouse NPC and neurons, and in human SH-SY5Y cells. Cut-off is Benjamini-Hochberg value < 0.05.

Supplementary Table 3

Genomic coordinates of the differentially enriched H3K9me2 peaks in the heads harvested from Day 50 male *NelfA*/+* flies and their control siblings; and their overlap with three subclasses of transposable elements.

Supplementary Table 4

Description of the primers used and their sequences

Supplementary experimental procedures

Fly Husbandry and genetics.

Flies were grown in standard fly media (6% cornmeal, 5% dextrose, 2.4% brewer's yeast, 0.8% agarose, 0.3% NIPAGIN) at 25°C with 12-hour light/dark cycle. To minimize the effect of circadian rhythm, fly tissues were harvested from 2 to 4 pm. *GeneSwitch* system (*GS*) was used for tissue-specific KD of various *NELF* genes (Osterwalder et al., 2001).

For generation of backcrossed *Nelf-A*^{KG09483}/+ line, PCR was conducted to validate the P-element integration site and the level of *Nelf-A* expression in the offspring.

RNA-sequencing and data processing

RNA-seq of biological replicates were conducted by BGI (Hong Kong) according to company protocol. RNA quality was assessed with Agilent 2100 bioanalyzer. Following DNAse I treatment, mRNA enriched by Oligo-dT coupled magnetic beads was fragmented and used as template to make cDNA library. Short DNA fragments were subjected to end-repair, A-tailing, adaptor ligation, size selection and sequenced on Illumina HiSeqTM4000.

The following methods were adapted from (Adusumalli et al., 2019). The quality of RNA-seq data was assessed by FastQC and the adaptor sequences were removed by TrimGalore. The reference genome of *D. melanogaster* (BDGP6.22.98) was indexed with STAR v2.5 using default parameters. RNA-seq reads were aligned to the indexed genome with STAR and the read counts were generated using HTseq-count. The differential gene expression between *NelfA*/+ flies* and their control siblings was identified by DESeq2, with heatmap generated using R Bioconductor. Gene Ontology analysis of differentially expressed genes (adjusted *p*-value < 0.05, baseMean > 500, $Log_2FC \geq \pm 0.3$) was performed using Database for Annotation, Visualization and Integrated Discovery (DAVID). Enriched biological processes with less than 0.05 *Benjamini-Hochberg* value are considered significant.

The abundance of transposable element (TE) transcripts was quantified by TEtranscripts (--mode multi) (2.1.4) (Jin et al., 2015). STAR was used to map the multi-reads with the maximum multiple alignments of not more than 100, using the following parameters --winAnchorMultimapNmax 100 --outFilterMultimapNmax 100. The aligned read counts were assigned to the corresponding TE based on TE annotation GTF file (dm6_BDGP_rmsk_TE.gtf.gz) and the differential expression analysis was performed using DESeq2.

Validation by Real-Time PCR

For validation of differential gene expression, total RNA was prepared from either 20 heads or 50 brains of adult flies. High capacity cDNA reverse transcription kit (Applied Biosystems) was used to generate cDNA libraries from 1 µg of total RNA pre-treated with DNase I (Thermo Scientific). For ChIP validation, DNA concentration was determined by Qubit fluorometer (Invitrogen). Site-specific primers

on *Hsp68* and *Hsp83* genes were designed based on RNAPII ChIP-seq data from KC167 cells (GSE116884) (Fig.S3B and S3C).

Real-time quantitative PCR was carried out in triplicates (6 µl/reaction) with 0.2 µM of primers and 2x SYBR green master mix (Thermo Scientific) using 7900HT Fast Real-Time PCR machine (Applied Biosystems). Relative gene expression was calculated using 2-ΔCT method where threshold cycle (CT) values of target genes were normalized to *act5C* gene.

Site-specific primers on *Hsp68* and *Hsp83* genes were designed based on RNAPII ChIP-seq data from KC167 cells KC167 cells (Nazer et al., 2018). For ChIP-qPCR, the CT value of H3K9me2 and RNAPII ChIP was calculated relative to its respective input DNA.

Antibody Generation

A 306 bp fragment from C-terminal region of *NELF-A* cDNA (SD07139, DGRC) was amplified by PCR (primers listed in Supplementary Table 4). After digestion by restriction enzymes, the purified PCR fragment was cloned into the *BamHI* and *XhoI* sites of pGEX-4T1 vector. Following verification by DNA sequencing, recombinant construct was transformed into BL21 competent cells for protein expression. GST-tagged NELF-A protein was purified by GST.Bind™ kits (Novagen) and used to generate antibody at Temasek Life Sciences Lab animal facility.

Immunoblot

Samples boiled in Laemmli buffer were resolved in 6-12% SDS-PAGE gel and subjected to standard western protocol. Transferred PVDF membranes were blocked in 5% milk or 5% BSA (in 0.1% Tween, PBS) and probed with the respective antibodies. Phospho-specific antibody to Histone 2A variant (γ-H2AV) was used to measure the level of DNA double-stranded breaks caused by H_2O_2 treatment (Lake et al., 2013).

Immunofluorescence

Heads harvested from Day 50 flies were fixed (4% formaldehyde, 0.1% Triton-X 100 in PBS) for 20 minutes at room temperature and washed three times with PBS. Brains were dissected from the heads in cold PBS and subjected to additional 15 minutes of fixation at room temperature. After three washes with PBS, the brains were first blocked (5% normal goat serum, 0.1% Triton-X 100 in PBS) for 30 minutes at room temperature, followed by overnight incubation with anti-H3K9me2 (ab1220) and anti-H3K9me3 (ab8898) antibodies (5µg/ml each in blocking buffer) at 4°C. After five rounds of PBS washes on the next day, the brains were incubated with anti-mouse 594 and anti-rabbit FITC antibodies (1:500, Invitrogen) in blocking buffer overnight at 4°C. Following three rounds of PBS washes, the brains were mounted in VECTASHIELD with DAPI (Vector labs) and Z-stack images across the entire

brain taken using confocal microscope (FV3000, 60X objective). The fluorescence intensity of H3K9me2/3 was quantified using ImageJ and normalized to DAPI signal.

Analysis of RNAPII ChIP-seq data from human SH-SY5Y cells

RNAPII ChIP-seq data with the following accession number GSM2864937, GSM2864938 and GSM1532411 were downloaded. The genomic coordinates of the MACS peaks from each dataset were overlapped using Galaxy to identify high-confidence RNAPII enrichment sites. Following gene annotation, GO analysis (geneontology.org using Reactome pathway with Fisher's Exact FDR) was conducted to characterize the highly paused genes in SH-SY5Y cells.

Chromatin Immunoprecipitation (ChIP) and sequencing

For each biological replicate, 100 fly heads were dounced 30 times with pestle B (Sigma) in 1 ml of 1% formaldehyde in A1 buffer (60 mM KCl, 15 mM NaCl, 4 mM $MgCl_2$, 15 mM HEPES pH 7.6, 0.5% NP-40, 0.5 mM DTT, 0.5 mM PMSF and protease inhibitors cocktail). The lysate was transferred to 1.5 ml eppendorf tube and incubated on nutator for 10 min at RT. Crosslinking was quenched by 5 minutes of incubation with glycine (final 125 mM) at room temperature. The supernatant was gently removed after 5 minutes of 4000 x g centrifugation at 4°C. After three additional rounds of A1 buffer wash and centrifugation, the nuclear pellet was resuspended with 0.6 ml of 1% RIPA buffer (10 mM Tris pH 8.0, 1 mM EDTA, 140 mM NaCl, 1% SDS, 0.1% sodium deoxycholate, 0.5 mM DTT, 0.5 mM PMSF, protease inhibitors cocktail). Total nuclear extract was sonicated using Bioruptor (30 sec ON/OFF, high settings, 30 cycles) to obtain DNA fragments of 200-700 bp. Following 10 minutes of 15,000 rpm centrifugation at room temperature, the supernatant was collected as the chromatin extract. One tenth of the chromatin extract was kept as input whereas the rest was diluted five-fold with 0.1% RIPA buffer (10 mM Tris pH 8.0, 1 mM EDTA, 140 mM NaCl, 1% Triton-X 100, 0.1% SDS, 0.1% sodium deoxycholate, 0.5 mM DTT, 0.5 mM PMSF, protease inhibitors cocktail) and incubated overnight with 5 μg of antibody (H3K9me2, ab1220 or total RNAP II, CTD4H8) at 4°C. On the following day, 20 μl of pre-blocked (0.5% BSA in PBS) Protein-A Sepharose beads (GE Healthcare) was added to the chromatin extract. Following 4 hours of incubation at 4°C, DNA-antibody-beads complex was centrifuged for 1 minute at 2000 x g to remove the supernatant. The beads complex was washed with 0.1% RIPA buffer for 5 times, LiCl buffer (10 mM Tris pH 8, 1 mM EDTA, 250 mM LiCl, 0.5% NP-40, 0.5% sodium deoxycholate) once and Tris-EDTA buffer (10 mM Tris pH 8, 1 mM EDTA) once. The DNA fragments were eluted twice from the beads, each time by incubating with 200 μl elution buffer (1 M $NaHCO_3$, 1% SDS) for 1 hour at 37°C. Input was topped up to 400 μl with elution buffer. After adding 20 μl 5 M NaCl, DNA was reverse crosslinked by overnight incubation at 65°C. On the next day, samples were treated with RNase A (20 μg/ml, 1 hour at 37°C) and proteinase K (40 μg/ml, 1 hour at 50°C). DNA was extracted by phenol/chloroform and ethanol precipitation. Barcoded libraries were generated for H3K9me2 ChIP DNA by KAPA real-time library amplification kit (KAPA

Biosystems) (Rao et al., 2020) and sequenced on Illumina Hiseq4000 platform (150 bp pair-end) at NovogeneAIT.

Cell culture

S2-DRSC cells (#181) were cultured in Schneider's Medium (Gibco, 21720001) supplemented with 10% FBS in 25°C.

SH-SY5Y cells (ATCC CRL-2266) were cultured in DMEM/F12 medium, supplemented with 10% fetal bovine serum, MEM non-essential amino acids and 1 mM sodium pyruvate (Gibco) at 37°C.

SAM assay

Bridge-It® *S*-adenosyl methionine (SAM) fluorescence assay kit (Mediomics®) was used according to the manufacturer's instructions. Briefly, 10 fly heads were homogenized in 50 μl of buffer CM and incubated for 1 hour at room temperature with occasional mixing by vortex. After 5 minutes of 10,000 x g centrifugation at 4°C, supernatant was collected and diluted 1:1 with Buffer S. 10 μl of the mixture was added to 90 μl SAM assay solution and incubated for 30 minutes at room temperature. To generate SAM standard curve, fluorescence was measured with excitation at 485 nm and emission at 665 nm. Relative fluorescence is calculated with the formula: RF = [(F-Fbg) – (Fo-Fb)] / Fo-Fb), according to manufacturer's protocol. SAM concentration for each sample was then determined with SAM standard curve.

H3K9me2 ChIP-seq data processing and analysis

The quality of the sequencing reads was assessed using FastQC and mapped to the *D. melanogaster* genome (BDGP6.22.98) using STAR (v2.5) with following parameters: (--alignEndsType EndToEnd --alignIntronMax 1). Bioinformatic analysis was performed on Galaxy platform. H3K9me2 enriched peaks were called using MACS2 (v2.1.1) with the following parameters: effective genome size of 120000000 --no model --extsize 200 --broad --keep-dup auto –bdg (Zhang et al., 2008). Differential peak analysis between *NelfA*/+* flies and their control siblings was performed using MACS2 bdgdiff with following parameters: -g 100 -l 200. This yielded three distinct groups, namely the shared common peaks (+/+ = *NelfA*/+*), increased enrichment in control siblings (+/+ > *NelfA*/+*) and higher enrichment in *NelfA*/+* flies (*NelfA*/+* > +/+). Bedtools intersect with default setting was used to overlap the shared and differential H3K9me2 peaks with curated TE GTF file. computeMatrix (v3.3.2) was used to build a matrix with a flanking region of ± 5 kb around TSS for H3K9me2 peaks that are highly enriched in *NelfA*/+* flies (*NelfA*/+* > +/+) and overlap with TEs. plotHeatmap (v3.3.2) was applied to generate heatmap and ChIPseeker (v1.18) used to annotate the enriched TE sites that overlapped with each group of H3K9me2 peaks. For Integrated Genome Viewer (IGV, v2.4), alignment reads BAM files were transformed into bigWig files of read coverage using bamCompare (v3.3.2) where the scores represent ChIP signal minus the Input.

Supplementary References

Adusumalli, S., Ngian, Z.K., Lin, W.Q., Benoukraf, T., and Ong, C.T. (2019). Increased intron retention is a post-transcriptional signature associated with progressive aging and Alzheimer's disease. Aging Cell *18*, e12928.

Jin, Y., Tam, O.H., Paniagua, E., and Hammell, M. (2015). TEtranscripts: a package for including transposable elements in differential expression analysis of RNA-seq datasets. Bioinformatics *31*, 3593-3599.

Lake, C.M., Holsclaw, J.K., Bellendir, S.P., Sekelsky, J., and Hawley, R.S. (2013). The development of a monoclonal antibody recognizing the Drosophila melanogaster phosphorylated histone H2A variant (gamma-H2AV). G3 (Bethesda) *3*, 1539-1543.

Nazer, E., Dale, R.K., Palmer, C., and Lei, E.P. (2018). Argonaute2 attenuates active transcription by limiting RNA Polymerase II elongation in Drosophila melanogaster. Sci Rep *8*, 15685.

Osterwalder, T., Yoon, K.S., White, B.H., and Keshishian, H. (2001). A conditional tissue-specific transgene expression system using inducible GAL4. Proc Natl Acad Sci U S A *98*, 12596-12601.

Rao, V.K., Swarnaseetha, A., Tham, G.H., Lin, W.Q., Han, B.B., Benoukraf, T., Xu, G.L., and Ong, C.T. (2020). Phosphorylation of Tet3 by cdk5 is critical for robust activation of BRN2 during neuronal differentiation. Nucleic Acids Res *48*, 1225-1238.

Zhang, Y., Liu, T., Meyer, C.A., Eeckhoute, J., Johnson, D.S., Bernstein, B.E., Nusbaum, C., Myers, R.M., Brown, M., Li, W., *et al.* (2008). Model-based analysis of ChIP-Seq (MACS). Genome Biol *9*, R137.
